# Zn^2**+**^ Coordination and Ligand-Exchange Dynamics in a ZnCl_2_‑Based Deep Eutectic Electrolyte from Machine-Learning Molecular Dynamics

**DOI:** 10.1021/acs.jpcb.6c04316

**Published:** 2026-09-26

**Authors:** Eudes Fileti, Davi Fileti, André P. L. de Carvalho, Débora Ariana C. da Silva, Shern Ren Tee, Dinis O. Abranches, João A. P. Coutinho

**Affiliations:** † Instituto de Ciência e Tecnologia, Universidade Federal de São Paulo, São José dos Campos, São Paulo 12247-014, Brazil; ‡ Institute of Mathematical Sciences and Computation, 28105 University of São Paulo, São Carlos, São Paulo 13566-590, Brazil; § Institute of Science and Technology, Federal University of ABC, Santo Andre, São Paulo 09210-170, Brazil; ∥ School of Environment and Science, 5723 Griffith University, Nathan, Queensland 4111, Australia; ⊥ CICECO − Aveiro Institute of Materials, Department of Chemistry, 426216 University of Aveiro, Aveiro 3810-193, Portugal

## Abstract

The Zn^2+^ coordination shell in ZnCl_2_-based battery electrolytes is far more dynamic than its time-averaged structure suggests. Using a machine-learning force field (MACE) fine-tuned with density functional theory data, we simulate nanosecond-scale molecular dynamics of a ZnCl_2_:urea:H_2_O deep eutectic electrolyte across 300–400 K, a time regime inaccessible to *ab initio* methods and poorly captured by classical force fields. The Zn^2+^ first coordination shell is tetrahedral and compositionally mixed with chloride ions, urea, and water contributing comparable shares. This overall occupancy remains essentially constant with temperature, masking markedly asymmetric underlying kinetics. Residence-time analysis reveals that chloride is essentially nonexchanging on the nanosecond time scale, while water and urea exchange continuously, with residence times an order of magnitude shorter and activation energies of 12.5 and 16.2 kJ/mol, respectively. Potential of mean force and speciation analyses identify ZnCl_2_ as the dominant solute species and show that ligand substitution proceeds predominantly through an associative pathway. Self-diffusion coefficients and activation energies for all four constituent species further establish a mobility hierarchy consistent with this coordination picture. Together, these results provide a molecular-level rationale for the transport behavior of ZnCl_2_-based electrolytes and a general framework for understanding ion speciation and lability in coordinating eutectic battery electrolytes.

## Graphical abstract

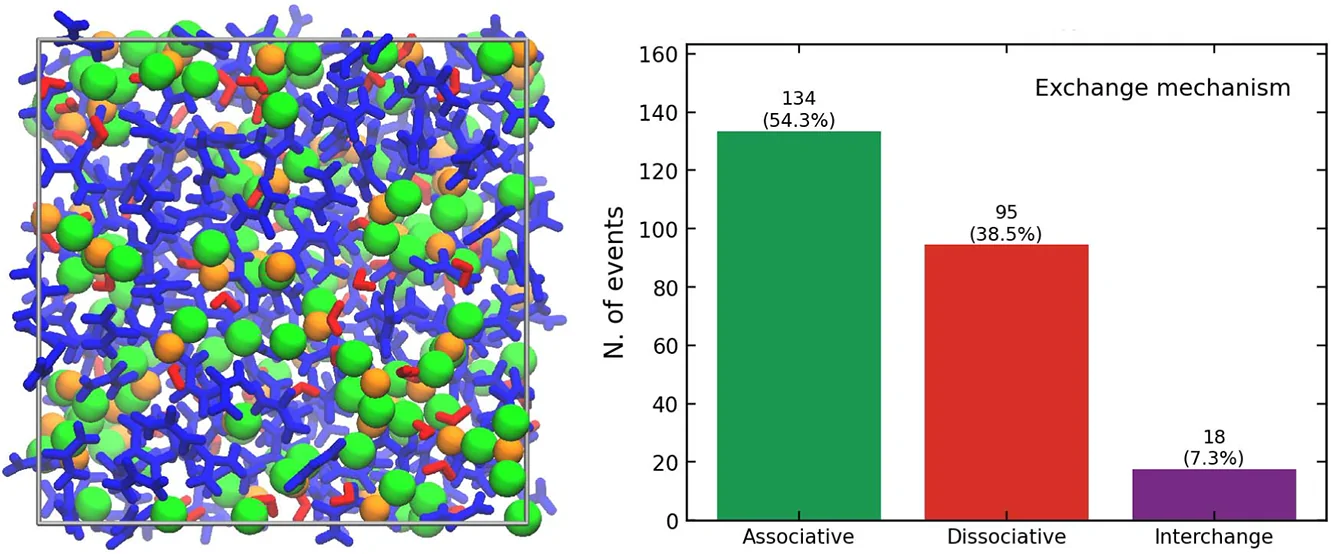

## Introduction

Zinc-based electrolytes have emerged as a particularly attractive platform for electrochemical energy storage devices, partly because metallic Zn is a promising anode material for batteries with aqueous electrolytes.^1–4^ However, its practical implementation is still limited by the high reactivity of free water and the parasitic reactions associated with Zn plating/stripping processes.^5^ In this context, highly concentrated electrolytes, eutectic mixtures, and “water-in-salt” or “water-in-DES” architectures have been extensively investigated as strategies to reduce water activity, modulate Zn^2+^ solvation, and improve the reversibility of reactions at Zn metal anodes.^1,5^ Zn-WO_3_ cells operating with 30 m ZnCl_2_ electrolytes have exhibited excellent capacity retention after 1000 cycles, and ZnCl_2_ in urea deep eutectic solvents has likewise been proposed as low-cost electrolytes for zinc-ion hybrid supercapacitors, reinforcing the technological relevance of this class of liquids for next-generation energy storage.^1^

ZnCl_2_/urea DES electrolytes occupy a particularly important position among Zn-based electrolytes because they combine a highly coordinated Zn environment with an intense, composition-sensitive hydrogen-bond network.^6,7^ Structural and periodic DFT studies on ZnCl_2_(urea)_2_ crystals indicate a tetrahedral Zn­(II) coordination environment with partially covalent Zn–Cl character, a feature invoked to rationalize the unusual fluidity and comparatively low ionic conductivity of these melts.^2^ The controlled addition of water adds a further layer of complexity: rather than acting as an inert diluent, water competes with chloride and urea for the Zn^2+^ coordination shell, reorganizing the local hydrogen-bond network and, in doing so, potentially modulating ionic transport and interfacial stability at the Zn anode.^8^

Beyond this static structural picture, the rate at which chloride, urea, and water exchange within the Zn^2+^ coordination shell is expected to govern key transport properties of these electrolytes. Mass spectrometry studies on ZnCl_2_/urea melts have identified a rich speciation landscape, including cationic complexes such as [ZnCl­(urea)]^+^ alongside anionic species such as ZnCl_3_^–^ and Zn_2_Cl_5_^–^, whose relative populations are thought to control ionic conductivity and viscosity.^2,9^ Such ensemble-averaged techniques, however, cannot resolve the underlying microscopic exchange events: which ligand is kinetically labile, how exchange proceeds mechanistically (for instance, through an associative or dissociative pathway), and how these factors respond to temperature and hydration remain open questions that are directly relevant to the rational design of Zn-electrolyte formulations for battery operation.

Capturing this competitive, fluxional coordination behavior is essentially out of reach for the methods conventionally available. Classical molecular dynamics simulations using fixed-charge force fields (CMD) cannot represent the electronic reorganization that accompanies Zn^2+^ ligand exchange, and polarizable force fields, while in principle better suited to this electronic detail, are themselves known to suffer from trajectory instabilities (“polarization catastrophe”)^10^ and a substantial parametrization burden in systems combining small, highly charged ions with extensive hydrogen-bond networks. Even AMOEBA, a polarizable force field developed specifically for divalent cations, has been shown not to adequately capture the charge-transfer character of the Zn–Cl interaction itself.^11,12^ *Ab initio* molecular dynamics (AIMD) simulations avoid these issues by construction but remain too costly to reach the nanosecond time scales over which ligand exchange occurs. This work would not have been possible without a method that closes that gap: we use a machine-learning force field (MACE), fine-tuned with density-functional-theory reference data, which combines near-quantum-mechanical accuracy with the nanosecond trajectories required to resolve ligand-exchange dynamics. Full validation of the model against *ab initio* benchmarks is provided in the Supporting Information.

In this work, we use nanosecond MACE-based machine-learning molecular dynamics (MLMD) simulation trajectories to characterize the coordination chemistry and ligand-exchange dynamics of Zn^2+^ in the ZnCl_2_:urea:H_2_O electrolyte across the 300–400 K range. We quantify the speciation of ZnCl_n_ complexes, the relative binding free energies of chloride, urea, and water at the Zn^2+^ center, and the residence times and exchange mechanisms that govern how these ligands interconvert. These results provide a molecular-level rationale for the transport and conductivity behavior of ZnCl_2_-based electrolytes and more broadly establish a framework for understanding ion speciation and lability in coordinating, eutectic battery electrolytes.

## Methodology

### System and Simulation Boxes

The system is a ZnCl_2_/urea/H_2_O deep eutectic electrolyte (DEE) with a 1:3:1 molar ratio (ZnCl_2_:urea:H_2_O). Ab initio reference trajectories were generated in a 491-atom cubic cell (17.86 Å edge). For MLMD, three system sizes were tested to assess finite-size effects on structure (see Figure 1): small (491 atoms, 17.9 Å, matching the AIMD cell), medium (982 atoms, 22.6 Å), and large (∼2000 atoms, 28.5 Å). The medium box was adopted for all production simulations as the best compromise between structural accuracy and computational cost. The cell edge of each box was fixed to reproduce the equilibrium density obtained from a 2 ns NPT equilibration at 300 K and 1 atm (ρ = 1.58 g/cm_3_); the AIMD cell and the three MLMD boxes therefore share a common number density, ensuring that the canonical-ensemble simulations sample the correct liquid density. Because the local speciation of concentrated Zn-halide systems can be sensitive to the initial arrangement of ions, several starting configurations were generated and independently equilibrated. The configuration whose first-shell speciation is consistent with experimental EXAFS^13^ data for aqueous ZnCl_2_ (Zn–Cl distance ≈2.25 Å, CN­(Zn–Cl) ≈2) and with recent MACE-based MLMD of aqueous ZnCl_2_ was retained for the production runs. The structural convergence of these trajectories is documented in the Supporting Information (Section S6). The large box, while useful for size-convergence testing, proved too computationally demanding for the 1 ns, five-temperature production protocol required for the dynamical analyses presented here.

**Figure 1:**
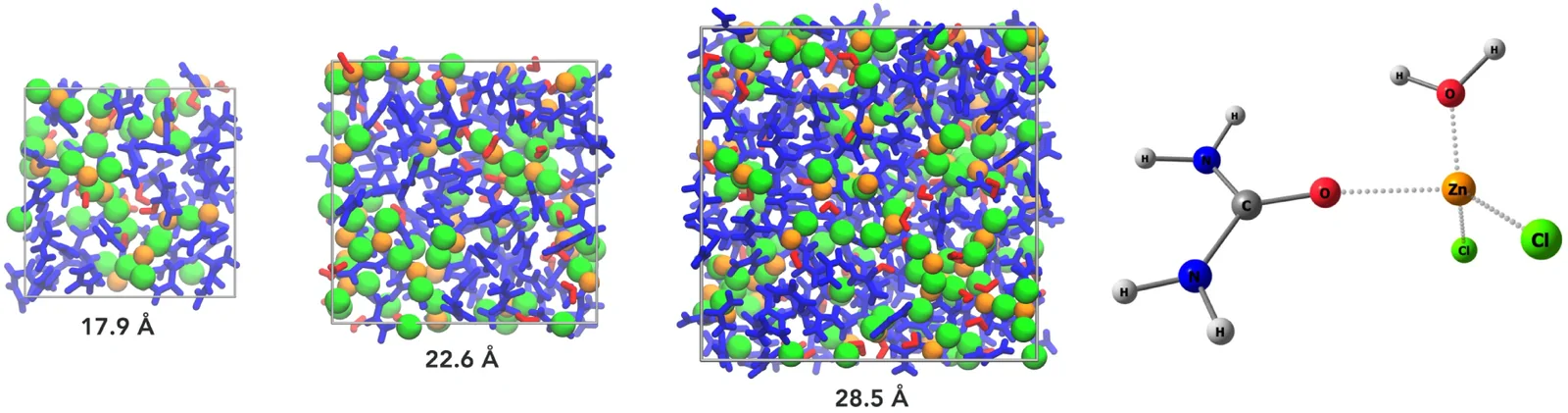
Representative simulation cells of the ZnCl_2_-based electrolyte with molar composition 1:3:1 (ZnCl_2_:urea:water), containing cubic boxes with edge lengths of 17.9, 22.6, and 28.5 Å. All systems were simulated using MLMD simulations, while the smallest cell was additionally investigated using AIMD simulations. The panel on the right shows a typical local coordination environment around Zn^2+^ (gold), where the metal ion is coordinated by two chloride ions (green), one urea molecule, and one water molecule (N: blue, H: light gray, C: dark gray, O: red).

We note that 400 K is adopted as a practical computational upper bound for the liquid range; all MLMD runs employ a fixed particle number (NVT), so evaporative water loss is not modeled. Experimental TGA data for this exact ternary composition are not available to us, and this temperature ceiling should therefore be read with the caveat that the stability of the mixture against water loss at 400 K has not been independently verified.

### Reference Data Set and Force Field Training

Density-functional-theory (DFT) reference data were generated with the CP2K^14^ package at the PBE-D3 level.^15,16^ Ten *ab initio* molecular dynamics (AIMD) trajectories of 6 ps (1 ps equilibration followed by 5 ps production) were generated at 300, 400, and 500 K using a multiseed protocol, and ∼10,000 configurations were reevaluated (energies and forces) at an elevated plane-wave cutoff of 650 Ry, to build a balanced training/test set of 2,800/600 configurations providing a good compromise between data diversity and computational cost. The MACE machine-learning force field was obtained by fine-tuning the MACE-MP-0 foundation model on this data set. Model performance was primarily assessed using the root-mean-square error (RMSE), the standard metric for this class of machine-learning potentials. The resulting model achieved validation RMSEs of 25 meV/Å for forces and 1.8 meV/atom for energies, values that are comparable to, or better than, those typically reported for machine-learning interatomic potentials developed for ionic liquids and deep eutectic electrolyte systems.^17,18^ Full DFT settings, data set construction protocol, and force field training hyperparameters are provided in the Supporting Information.

### CMD Simulations

CMD simulations were carried out with GROMACS^19^ for comparison with the results of the AIMD and MLMD simulations. The configuration was assembled using the PACKMOL^20^ package considering a cubic box with edges of 4.5 nm. All potential models used to describe intermolecular interaction were based on the GAFF-D3 force field.^21^ The water molecule was treated using the SPC/E model.^22^ The equilibration of the DEE was performed in an isothermal–isobaric ensemble (NPT) with T = 300 K and p = 1 atm for 2 ns, and the production was performed using the canonical ensemble (NVT) for 30 ns. A Parrinello–Rahman barostat^23^ was utilized to maintain constant pressure with a time constant of 2 ps and compressibility constant of 4.5 × 10^–5^ bar^–1^. This value corresponds to pure water; the isothermal compressibility of the ternary DES is expected to differ, but the Parrinello–Rahman barostat is robust to this parameter, which controls only the rate of volume relaxation rather than the equilibrium properties of the sampled ensemble. To ensure a constant temperature, a Nosé–Hoover thermostat^24^ was used with a time constant of 2 ps (the same as used in the NVT simulations). The Lennard-Jones interaction computed by the classical shifted force technique and long-range electrostatic correction estimated by the Particle Mesh Ewald method (PME)^25^ were applied beyond a cutoff radius of 0.9 nm. Periodic boundaries were employed in all directions.

### MLMD Simulations

Production MLMD simulations were performed with LAMMPS interfaced to the trained MACE potential via the ACEsuit package,^26^ using a 1 fs time step and three-dimensional periodic boundary conditions. After energy minimization and a 50 ps NVT equilibration (Nosé–Hoover thermostat), 1 ns production trajectories were generated independently at five temperatures (300, 325, 350, 375, and 400 K) spanning the liquid range of the electrolyte. Structural and dynamical properties (radial distribution functions, coordination numbers, mean-square displacements, and dimer existence autocorrelation functions for residence-time analysis) were computed from the production trajectories using the TRAVIS package.^27^ Structural convergence of all production trajectories was verified by monitoring the time evolution of the Zn^2+^ coordination numbers and the ionic-aggregate count (Supporting Information, Section S6). Full simulation protocol details (thermostat parameters, dump frequency, momentum correction, hyperparameters, models, and data split) are provided in the Supporting Information.

## Results and Discussion

### Zn^2+^ Coordination Environment and Its Thermal Response

Full validation of the MACE potential, including its agreement with AIMD for all pair correlations, the comparison against a classical force field, and the assessment of finite-size effects, is provided in the Supporting Information; here, we focus on the structure and coordination chemistry of the Zn^2+^ center, which is most directly relevant to the electrochemical behavior of this electrolyte.

Figure 2 compares the radial distribution functions (RDF) obtained from CMD, AIMD, and MLMD at 300 K. For all Zn^2+^-centered pairs (panels a–c), AIMD and MLMD curves overlap almost exactly: the Zn–Cl, Zn–O_w_, and Zn–O_u_ correlations each show a single, sharp first peak (near 225, 202, and 198 pm, respectively) followed by a deep minimum, the hallmark of a well-defined, exchange-limited first coordination shell. The Cl-centered and water oxygen–oxygen correlations (panels d–f) show good agreement between AIMD and MLMD, including the position and relative intensity of second-shell features. Notably, the Cl–N­(urea) correlation (panel e), which reflects the N–H···Cl^–^ hydrogen-bond geometry, is well-reproduced by the MACE model, with a first-shell peak at 342 pm consistent with moderate-strength hydrogen bonding. For this pair, the classical force field also captures the peak position reasonably well, in contrast to the urea–urea and urea–water correlations where CMD substantially overstructures relative to AIMD. The classical force field, by contrast, reproduces the general peak positions but fails qualitatively for the hydrogen-bonded urea network: CMD substantially overstructures the urea–urea and urea–water correlations relative to AIMD, and this is attributable to the absence of explicit polarization and charge transfer in the fixed-charge models considered.

**Figure 2:**
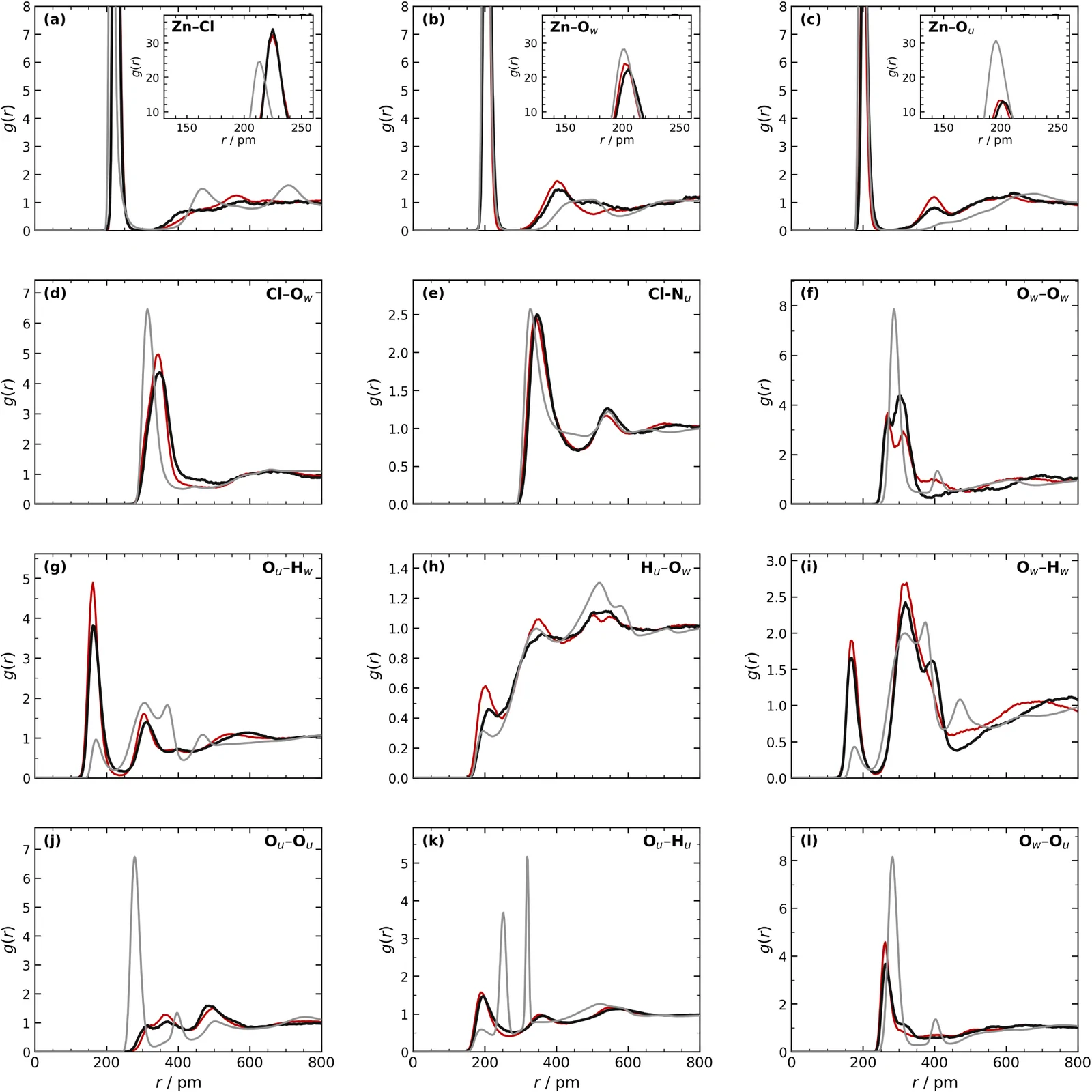
Radial distribution computed from classical molecular dynamics (CMD, gray), *ab initio* molecular dynamics (AIMD, black), and machine-learning molecular dynamics using the MACE force field (MLMD, red) for the ZnCl_2_:urea:H_2_O (1:3:1) deep eutectic electrolyte at 300 K. Panels (a)–(c) show Zn^2+^-centered pairs; insets highlight the sharp first-coordination-shell peak. Panels (d)–(f) show Cl^–^-centered and water oxygen–oxygen pairs. AIMD and MLMD use the same box size, 491 atoms. The close agreement between AIMD and MLMD curves across all pair types validates the MACE model for structural properties of this electrolyte.

These qualitative observations are confirmed quantitatively in Table 1. MLMD and AIMD agree closely on the composition of the Zn^2+^ first coordination shell: CN­(Zn–Cl) = 2.04 vs 2.06, CN­(Zn–O_w_) = 0.64 vs 0.75, and CN­(Zn–O_u_) = 1.35 vs 1.19, giving a total coordination number of 4.03 versus 4.00 and confirming a tetrahedral, 4-fold coordinated Zn^2+^ center in which chloride accounts for about half of the total occupancy, with the remainder partitioned between water and urea oxygen donors. This three-way partitioning of the first shell is consistent with the rich speciation landscape reported by mass spectrometry studies of ZnCl_2_/urea melts, where cationic [ZnCl­(urea)]^+^ and related mixed-ligand complexes coexist with more chlorinated species and the recovered CN­(Zn–Cl) ≈2 parallels the [ZnCl_2_]-type core motif observed in that work. The tetrahedral geometry itself mirrors the Zn­(II) environment reported crystallographically for anhydrous ZnCl_2_(urea)_2_,^2^ indicating that the local coordination preference of Zn^2+^ persists from the anhydrous DES into the hydrated electrolyte studied here, even as water displaces part of the urea population. The model also reproduces the hydrogen-bonding organization beyond the immediate Zn^2+^ shell, with CN­(Cl–N_u_) = 4.8 which reflects the number of urea N–H donors surrounding each chloride and CN­(O_u_–H_u_) = 1.58 vs 1.90.

**Table 1:** Structural Parameters of the First Coordination Shell for Selected Atom Pairs in ZnCl_2_:Urea:Water (1:3:1) at 300 K, Computed from AIMD and MLMD Simulations^a^

	**AIMD**			**MLMD**		
**Pairs**	**r**_**peak**_	**r**_**min**_	**CN**	**r**_**peak**_	**r**_**min**_	**CN**
**Zn–Cl**	225	288	2.1	225	295	2.0
**Zn–O**_**w**_	205	262	0.8	202	268	0.6
**Zn–O**_**u**_	202	268	1.2	198	268	1.4
**Zn**_**(total)**_			4.1			4.0
**Cl–N**_**u**_	345	412	4.8	342	410	4.8
**O**_**u**_ **–H**_**u**_	195	275	1.9	188	268	1.6

a The coordination number (CN) is defined as the running integral of the RDF, evaluated at the first minimum r_min_ of each pair correlation: $CN\left(r\right)=4\pi \rho \int_{0}^{r}g\left(r^{’}\right)r^{’2}dr^{’}$ where *ρ* is the bulk number density. Note that for Cl–N_u_, the broad first shell reflects N–H···Cl^–^ hydrogen-bond geometry rather than direct ionic coordination. Zn_(total)_ refers to the sum of all oxygen and chloride contributions to the Zn^2+^ first shell.

The coordination of Cl^–^ is markedly lower than the hydration number of ∼7 reported for chloride in dilute aqueous solution. This is a direct consequence of composition and speciation: with two chlorides per zinc and CN­(Zn–Cl) ≈2.0, essentially every Cl^–^ is bound within a Zn^2+^ first shell, where it is screened from the solvent, and the surrounding medium is urea-rich and water-poor (one H_2_O per two Cl^–^). The reduced coordination therefore reflects the ion-rich structure of the DES rather than a simulation artifact, and the recovered Zn–Cl distance (225 pm) agrees quantitatively with EXAFS^13^ and with MACE-based MLMD of aqueous ZnCl_2_.^4^

This coordination pattern remains remarkably stable across the accessible liquid range. As shown in Figure 3, CN­(Zn–Cl) decreases only marginally, from 2.04 to 2.00, between 300 and 400 K, while the total Zn^2+^ coordination number stays essentially constant (∼4.0). The composition of the oxygen-donor population, however, changes: CN­(Zn–O_u_) increases from 1.35 to 1.48 with temperature, at the expense of CN­(Zn–O_w_), suggesting the influence of temperature over oxygen’s vibrations. This compositional shift indicates a progressive, modest preference of Zn^2+^ for urea over water as temperature increases, even though the total first-shell occupancy is conserved. Taken together with the near-invariant Zn–Cl occupancy, these results show that the average coordination number of Zn^2+^ is a poor proxy for the underlying dynamics of the system: as we show in the following section, this apparently rigid, thermally robust shell is in fact the time average of a continuously fluctuating population of bound and unbound ligands, whose individual residence times differ by more than an order of magnitude.

**Figure 3:**
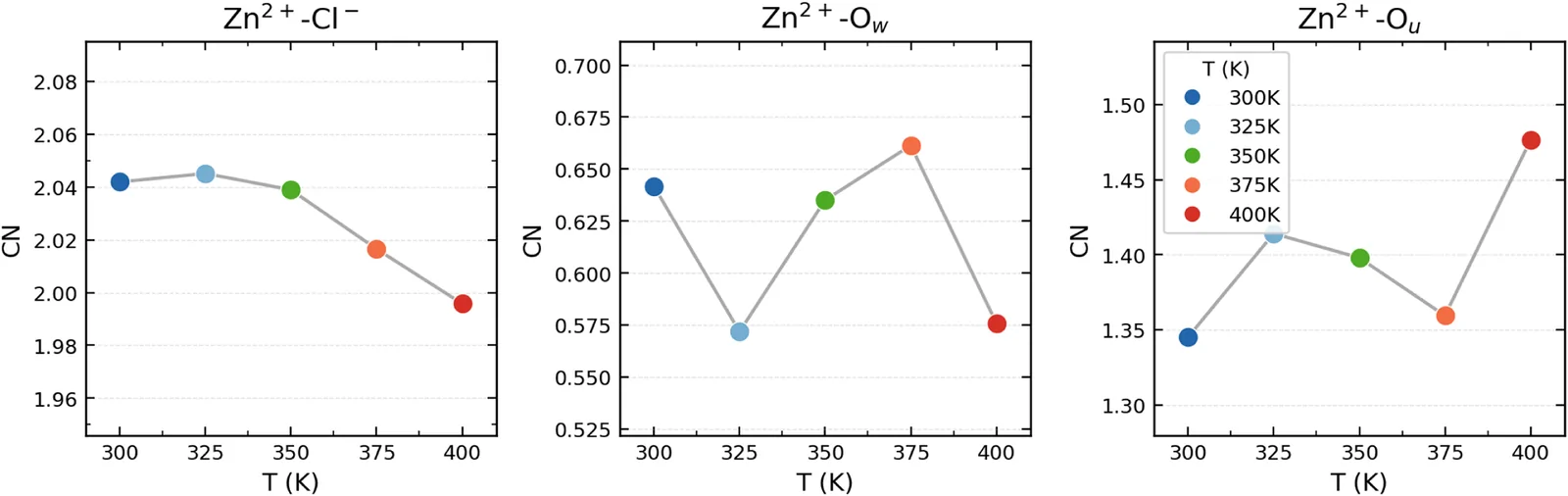
Coordination numbers CN­(Zn^2+^–Cl^–^), CN­(Zn^2+^–O_w_), and CN­(Zn^2+^–O_u_) as a function of temperature, extracted by integrating the corresponding RDFs up to the first minimum. The Zn^2+^ first-coordination shell retains a nearly constant total occupancy across 300–400 K, with a slight increase in urea coordination at the expense of water and chloride at elevated temperatures.

### Temperature-Dependent Diffusion

The structural picture established by RDF and CN analysis, while essential, provides only a static snapshot of the electrolyte. The dynamical behavior, how fast ions and solvent molecules migrate through the medium, is equally critical for understanding the electrochemical performance of the DEE, yet it is precisely in this regime that both AIMD and CMD fall short: AIMD trajectories are too short (∼50–100 ps) to reach the diffusive regime for sluggish species such as Zn^2+^, while CMD potentials lack the electronic polarization needed to capture the strong, directional coordination that governs ion mobility in this system. The 1 ns MLMD trajectories reported here bridge this gap, granting access to well-converged diffusion coefficients and activation energies for all four constituent species simultaneously (Figure 4).

**Figure 4:**
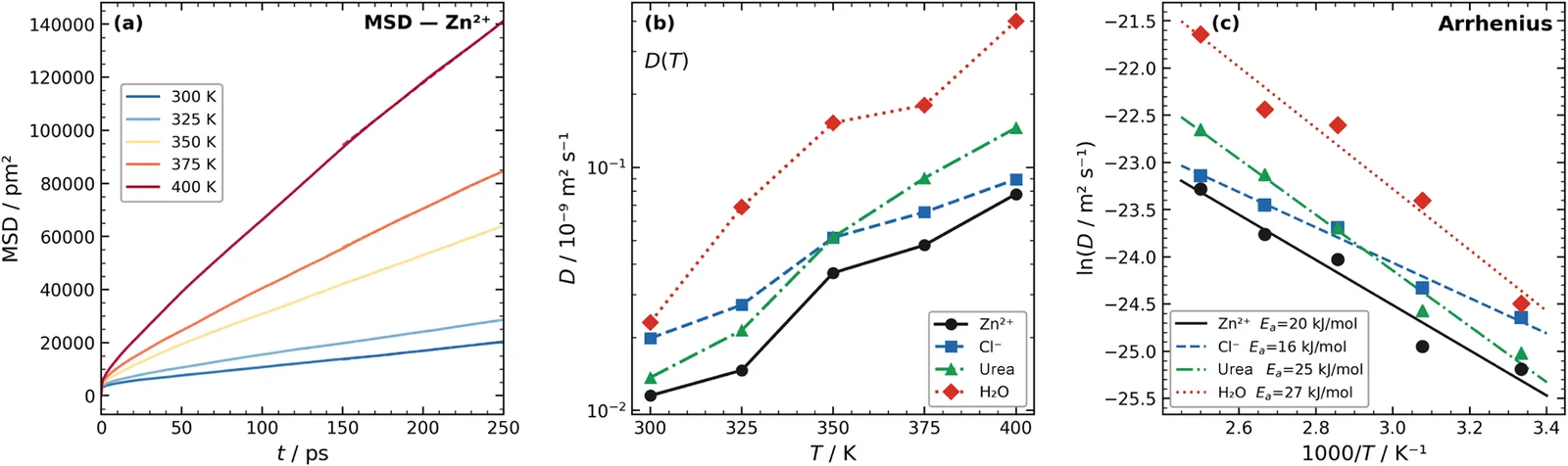
Dynamical properties of the ZnCl_2_:urea:H_2_O (1:3:1) DEE computed from 1 ns MLMD trajectories (medium simulation box, 982 atoms). (a) Mean-square displacement (MSD) of Zn^2+^ as a function of lag time at five temperatures (300–400 K). Solid lines: MSD curves; dashed lines of matching color: linear regression fits used to extract the diffusion coefficient D. (b) Self-diffusion coefficients D (log scale) as a function of temperature for all species. The mobility hierarchy (H_2_O > urea ∼Cl^–^ > Zn^2+^) reflects the distinct coordination environments of each species. (c) Arrhenius plot of ln­(D) versus 1000/T for the four species. Symbols: computed D values; lines: linear fits from which the activation energies *E*_a_ are extracted (see text).

The mean-square displacement (MSD) of each species was computed from the center-of-mass trajectories of the molecules/ions using the TRAVIS analyzer.^27^ For each temperature, the MSD was evaluated up to a correlation depth of 30% of the total trajectory length; a standard precaution that ensures adequate statistical averaging while avoiding the noise-dominated tail of the MSD; and the self-diffusion coefficient D was extracted from the linear (Einstein) regime by least-squares regression; full fitting parameters (correlation depth, regression window) are given in the Supporting Information. The complete set of MSD curves for all species and temperatures is presented in Figure S6, and the resulting diffusion coefficients are collected in Table S1 and plotted in Figure 4. A representative subset of the Zn^2+^ MSD curves is shown in Figure 4a; all of them display the expected ballistic-to-diffusive crossover, confirming that the 1 ns trajectories reach true Fickian diffusion at every temperature studied. The diffusion coefficients increase monotonically with temperature for all species and display a clear, consistent mobility hierarchy (Table S1): H_2_O is consistently the most mobile constituent (0.399 × 10^–9^ m^2^ s^–1^ at 400 K), followed by urea (0.145 × 10^–9^ m^2^ s^–1^) and Cl^–^ (0.089 × 10^–9^ m^2^ s^–1^), with Zn^2+^ being the least mobile species (0.078 × 10^–9^ m^2^ s^–1^ at 400 K). This ordering is consistent with the coordination environments identified from the RDF analysis: Zn^2+^ is strongly coordinated by ∼2 Cl^–^ and ∼1.4 urea oxygen atoms in a tight first shell, imposing a large effective hydrodynamic radius and correspondingly slow dynamics. Above 350 K, urea diffuses faster than Cl^–^, likely because urea, despite participating in the Cl^–^···H–N hydrogen-bond network, retains rotational and conformational degrees of freedom that accelerate its translational diffusion relative to the more isotropically solvated chloride anion. Water, present in a minority ratio (H_2_O:urea = 1:3) and less constrained by the ionic coordination network, retains markedly higher mobility at all temperatures, reaching diffusivities approximately five times larger than Zn^2+^ at 400 K.

The activation energies extracted from the Arrhenius fits (Figure 4c) are 19.9, 15.6, 24.6, and 26.9 kJ mol^–1^ for Zn^2+^, Cl^–^, urea, and H_2_O, respectively. The lowest *E*_a_ belongs to Cl^–^ (15.6 kJ mol^–1^), consistent with its relatively labile and geometrically flexible first solvation shell. Zn^2+^ presents an intermediate value (19.9 kJ mol^–1^) that reflects the energetic cost of partial desolvation of its tight first coordination shell during translational motion. The highest activation energies belong to urea (24.6 kJ mol^–1^) and H_2_O (26.9 kJ mol^–1^), reflecting the energetic cost of disrupting the extended hydrogen-bond network in which these species are embedded before translational diffusion can occur. The ordering *E*_a_(Cl^–^) < *E*_a_(Zn^2+^) < *E*_a_(urea) < *E*_a_(H_2_O) thus reflects a hierarchy of structural constraints: ionic coordination for the cation and anion and hydrogen-bond network disruption for the molecular species.

### Ligand-Exchange Dynamics at the Zn^2+^ Coordination Center

The coordination numbers discussed above are time-averaged occupancies; they say nothing about how long an individual ligand remains bound to Zn^2+^ or by what pathway exchange occurs. To resolve this kinetic dimension, we computed the dimer existence autocorrelation function (DACF) for each Zn–ligand pair, from which residence times and exchange mechanisms were extracted. Figure 5 brings together five complementary lines of evidence: the raw decay of ligand association (Figure 5a), the resulting residence times and their temperature dependence (Figure 5b,c), the underlying binding energetics (Figure 5d), the population of Zn-Cl_n_ species (Figure 5e), and the dominant exchange pathway (Figure 5f).

**Figure 5:**
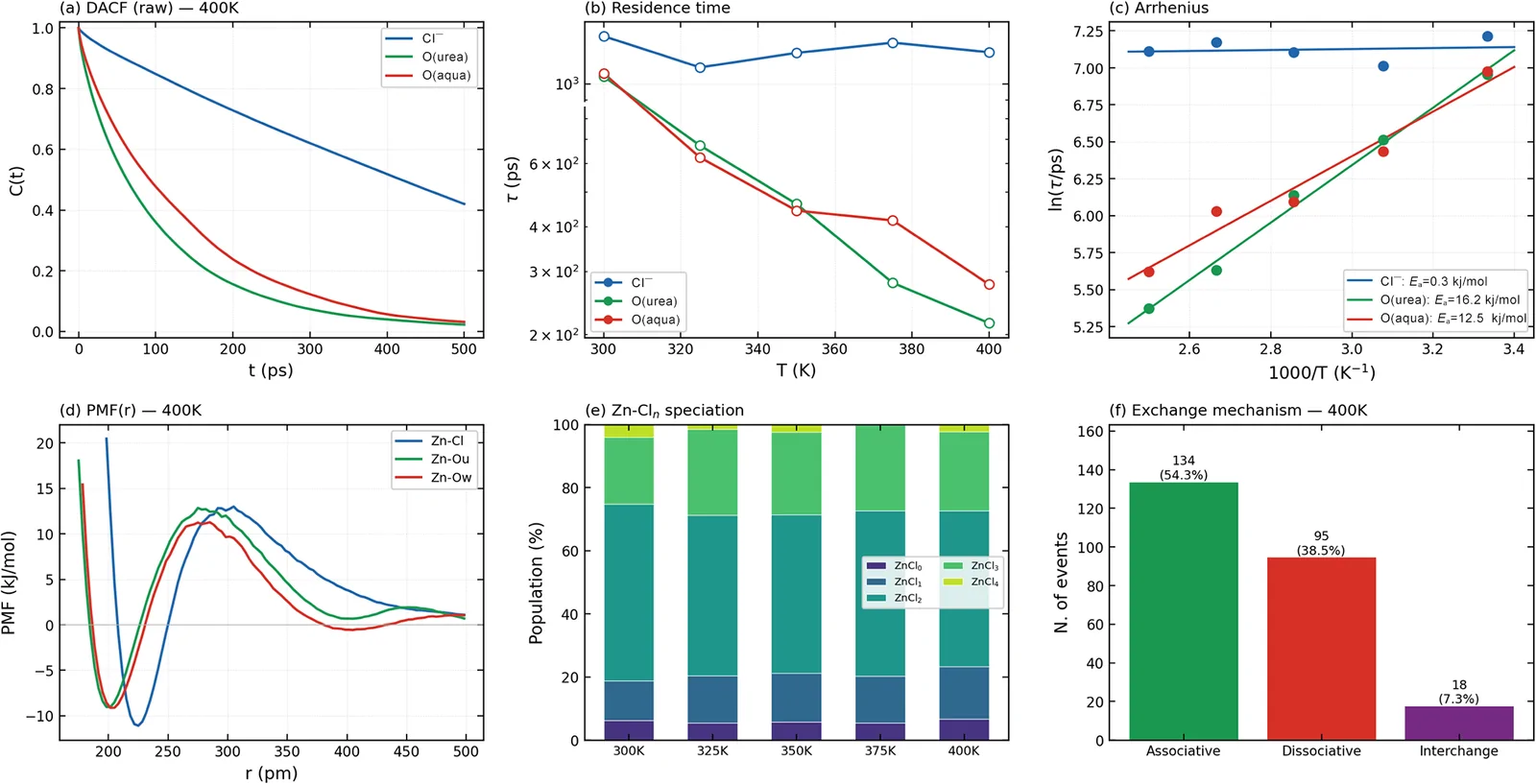
Ligand-exchange dynamics at the Zn^2+^ coordination center in the ZnCl_2_:urea:H_2_O (1:3:1) deep eutectic electrolyte, computed from 1 ns MLMD trajectories (medium box, 982 atoms). (a) Raw dimer existence autocorrelation functions (DACF) for Zn–Cl, Zn–O­(urea), and Zn–O­(water) at 400 K, using cutoff distances of 295, 250, and 260 pm, respectively. (b) Residence times τ, extracted from polyexponential fits to the DACF, as a function of temperature; open symbols (Zn–Cl) denote temperatures at which the DACF had not decayed sufficiently within the 500 ps correlation window for a reliable extrapolation, so these values represent lower bounds. (c) Arrhenius plot of ln­(τ) versus 1000/T, with activation energies *E*_a_ obtained by linear regression. (d) Potentials of mean force, PMF­(r) = −RT ln g­(r), at 400 K for the same three pairs. (e) Population of Zn-Cl_n_ (n = 0–4) species as a function of temperature, from graph-based clustering of the Zn–Cl coordination network. (f) Classification of ligand-exchange events into associative, dissociative, and interchange pathways at 400 K, after applying a 2 ps minimum-dwell filter to suppress transient recoordination noise.

At 400 K, the raw DACF curves (Figure 5a) already convey the central qualitative result: Cl^–^ remains associated with Zn^2+^ for most of the 500 ps correlation window, decaying only to C­(t) ≈0.43, whereas both oxygen donors decay to near zero within the same window, with O­(water) lagging slightly behind O­(urea). This order-of-magnitude separation between an essentially nonexchanging chloride and two comparatively labile oxygen donors is the qualitative signature that the quantitative analysis below makes precise.

The temperature dependence of the extracted residence times (Figure 5b) confirms this picture. At 300 K, all three ligands reside in a comparable range, between ∼1050 and ∼1360 ps, with Zn–Cl already the longest-lived. As the temperature increases to 400 K, τ­(O–urea) and τ­(O–water) decrease roughly 5-fold, to 215 and 276 ps, respectively, while τ­(Zn–Cl) shows no systematic decrease, fluctuating between ∼1100 and ∼1600 ps with no clear trend. Arrhenius analysis of the two oxygen donors (Figure 5c) yields activation energies of 16.2 kJ/mol for O­(urea) and 12.5 kJ/mol for O­(water); the lower value for water indicates that it is the more kinetically labile ligand, consistent with its slightly longer, weaker Zn–O bond relative to the more strongly Lewis-basic urea carbonyl oxygen. The corresponding fit for Zn–Cl gives a formally negligible activation energy (0.3 kJ/mol), but this should not be read as evidence of temperature-insensitive binding: because τ­(Zn–Cl) exceeds the simulation window at every temperature studied, the DACF never decays enough for the extrapolated integral to be statistically reliable, and the apparent flatness instead reflects this sampling limitation. What can be stated with confidence is that chloride remains essentially nonexchanging on the nanosecond time scale accessible here, at all five temperatures. We note that, precisely because chloride exchange is slow relative to the accessible time scale, the Zn^2+^ first shell relaxes only gradually at the lowest temperatures. A structural-convergence analysis (Supporting Information, Section S6) confirms that the coordination environment is stationary to within 2% at 375–400 K and evolves slowly, through physical ligand exchange rather than numerical drift, at 300–350 K, so that the dynamical properties reported here are unaffected by this low-temperature relaxation.

The potentials of mean force (Figure 5d, shown at 400 K) provide the energetic basis for this kinetic hierarchy. The Zn–Cl well is the deepest of the three (−11.1 kJ/mol), followed by Zn–O_w_ (−9.1 kJ/mol) and Zn–O_u_ (−9.0 kJ/mol); the marginally deeper Zn–O_w_ well is consistent with its marginally longer residence time relative to that of Zn–O_u_. The barrier separating the first and second coordination shells is correspondingly higher for Zn–Cl than for either oxygen donor, in qualitative agreement with its much longer residence time. These barrier heights are, however, systematically larger than the activation energies obtained from the dynamic DACF analysis; this is expected since a one-dimensional radial PMF is known to overestimate the true, multidimensional kinetic barrier for ligand exchange, which can proceed via lower-energy angular or concerted pathways not captured by a purely radial coordinate. PMF curves are shown at a single reference temperature because the apparent deepening of the well with temperature in PMF units is largely a consequence of the explicit RT prefactor rather than genuine structural reorganization (Supporting Information).

The speciation analysis (Figure 5e) identifies ZnCl_2_ as the dominant species at all temperatures (49.5–56.1% population), in agreement with CN­(Zn–Cl) ≈2 and with the [ZnCl_2_]-type core motif inferred from mass-spectrometric speciation studies of ZnCl_2_/urea melts. A substantial fraction of Zn^2+^ centers, however, exist in bridged, multi-Zn clusters connected through shared chloride ions; this fraction decreases markedly with temperature, from 34.3% at 300 K to approximately 13% at 400 K, indicating that thermal agitation progressively breaks up chloride-bridged aggregates in favor of more isolated, mononuclear ZnCl_2_ units. To resolve the instantaneous Zn^2+^ environment beyond the chloride count, each Zn^2+^ center was assigned at every trajectory frame to a coordination motif ZnCl_*x*_O­(U)_*y*_O­(W)_*z*_, defined by the number of Cl^–^, O­(urea), and O­(water) donors within the first shell (cutoffs of 295, 250, and 260 pm, respectively); the results are shown in Figure S8. The dominant motif at all temperatures is ZnCl_2_O­(U)­O­(W) (a mixed tetrahedral environment with two chlorides and one donor each from urea and water) coexisting with ZnCl_2_O­(U)_2_ and ZnCl_3_O­(U) as major secondary species. The motif populations shift systematically with temperature: water-containing species decrease (ZnCl_2_O­(W)_2_: 6.5 → 2.1%) while urea-rich and chloride-rich species increase (ZnCl_2_O­(U)_2_: 14.3 → 19.4%; ZnCl_3_O­(W): 3.3 → 12.6%), in direct correspondence with the CN­(Zn–O_w_) and CN­(Zn–O_u_) trends of Figure 3.

Finally, classification of the individual exchange events recorded in the DACF trajectories (Figure 5f) shows that ligand substitution at Zn^2+^ proceeds predominantly through an associative pathway (54.3% of events), in which the incoming ligand coordinates before the outgoing one fully departs, transiently raising the local coordination number above four. Dissociative events, where a ligand departs before a replacement arrives, account for a substantial secondary fraction (38.5%), while interchange pathways with no resolvable intermediate (7.3%) are comparatively rare. The dominance of the associative mechanism is consistent with the slight, temperature-driven increase in urea occupancy noted above: rather than first vacating a site, incoming urea molecules appear to displace water and chloride from an already occupied shell.

## Conclusions

Using a MACE machine-learning force field trained on density-functional-theory data, we characterized the structure and dynamics of a ZnCl_2_:urea:H_2_O deep eutectic electrolyte at the molecular level over nanosecond time scales inaccessible to *ab initio* simulation. The Zn^2+^ center adopts a tetrahedral, 4-fold coordination shell shared among chloride, urea, and water, whose total occupancy is essentially temperature-invariant; however, this apparent rigidity conceals pronounced kinetic asymmetry. Chloride remains bound to Zn^2+^ for the full duration of our trajectories, while water and urea exchange on the 200–1000 ps time scale with modest activation energies, predominantly through an associative mechanism in which the incoming ligand displaces an existing one rather than waiting for a vacancy. Speciation analysis identifies ZnCl_2_ as the dominant solute species, coexisting with a population of chloride-bridged multi-Zn clusters that diminishes with temperature. Resolving the instantaneous coordination environment further shows that ZnCl_2_O_u_O_w_, a mixed tetrahedral motif with one urea and one water donor, is the single most populated species at all temperatures and that the motif distribution shifts progressively from water-containing to urea- and chloride-rich environments as temperature increases. Diffusion coefficients for all four species establish a mobility hierarchy (H_2_O > urea > Cl^–^ > Zn^2+^) that is directly consistent with this coordination and exchange picture: species more tightly and more persistently bound to Zn^2+^ diffuse more slowly. Together, these results show that the transport properties of ZnCl_2_-based electrolytes cannot be rationalized from time-averaged structures alone and that ligand lability (not just ligand identity) is a key variable for the rational design of Zn-battery electrolyte formulations. Extending this approach to other compositions and metal-halide chemistries is a natural next step toward a predictive, molecular-level framework for eutectic battery electrolytes.

## Supplementary Material



## Notes

DFT settings, data set construction, and MACE training hyperparameters; validation of the MACE force field against AIMD and CMD, including finite-size effects; radial distribution functions; potentials of mean force at all temperatures; and mean-square-displacement curves, self-diffusion coefficients, and coordination-motif populations versus temperature (PDF)

## References

[1] Chen D., Wang Y., Yao T., Yang H., Shen L. (2025). Unraveling
the Exceptional Kinetics of Zn||organic Batteries in Hydrated Deep
Eutectic Solution. J. Energy Chem.

[2] Malinowski A., Śmiechowski M. (2024). Computational
Study of Molecular Interactions in ZnCl2­(Urea)­2
Crystals as Precursors for Deep Eutectic Solvents. Crystals.

[3] Hao J., Zhang S., Wu H., Yuan L., Davey K., Qiao S.-Z. (2024). Advanced Cathodes for Aqueous Zn Batteries beyond Zn ^2+^ Intercalation. Chem. Soc. Rev.

[4] Brahma D., Avula N. V. S., Balasubramanian S. (2026). Hydration
Environment of Complexes
and Their Dynamics in Aqueous Zinc Chloride Solutions. J. Phys. Chem. B.

[5] Wang L., Deng Y., Yan L. (2023). Low-Cost “Water-in-Deep Eutectic
Solvent” Electrolyte for High-Performance Zinc Ion Hybrid Supercapacitors. ACS Appl. Energy Mater.

[6] Ou Z., Zhao M., Zhu K., Liu Z., Yang S., Zhang H., Wang F., Yang W. (2026). Constructing
Robust
Hydrogen Bond Networks in Electrolytes for Long-Life Zinc-Ion Batteries. Angew. Chem., Int. Ed.

[7] Xiao Z., Dai X., Zhu J., Liu D., Liu L., Liu X., Li Y., Qian Z., Wang R. (2025). Hydrogen Bond Competition Optimizing
Aqueous Zn Ion Solvation and (002) Interfacial Deposition with Ultralong
Stability. Adv. Funct. Mater.

[8] Sethi A., Rajeev C., Anil Kumar U., Sharma P., Sunda A. P., Dhavale V. M. (2026). Effect of Anions
in Zn-Based Aqueous Electrolyte on
Electrochemical Performance of Prussian Blue Analogue. Batteries Supercaps.

[9] Abbott A. P., Barron J. C., Ryder K. S., Wilson D. (2007). Eutectic-Based Ionic
Liquids with Metal-Containing Anions and Cations. Chem. -Eur. J.

[10] Goloviznina K., Gong Z., Costa Gomes M. F., Pádua A. A. H. (2021). Extension
of the CL&Pol Polarizable Force Field to Electrolytes, Protic
Ionic Liquids, and Deep Eutectic Solvents. J.
Chem. Theory Comput.

[11] Duboué-Dijon E., Mason P. E., Fischer H. E., Jungwirth P. (2018). Hydration
and Ion Pairing in Aqueous Mg ^2+^ and Zn ^2+^ Solutions:
Force-Field Description Aided by Neutron Scattering Experiments and
Ab Initio Molecular Dynamics Simulations. J.
Phys. Chem. B.

[12] Wu J. C., Piquemal J.-P., Chaudret R., Reinhardt P., Ren P. (2010). Polarizable Molecular Dynamics Simulation
of Zn­(II) in Water Using
the AMOEBA Force Field. J. Chem. Theory Comput.

[13] D’angelo P., Zitolo A., Ceccacci F., Caminiti R., Aquilanti G. (2011). Structural
Characterization of Zinc­(II) Chloride in Aqueous Solution and in the
Protic Ionic Liquid Ethyl Ammonium Nitrate by x-Ray Absorption Spectroscopy. J. Chem. Phys.

[14] Kühne T. D., Iannuzzi M., Del Ben M., Rybkin V. V., Seewald P., Stein F., Laino T., Khaliullin R. Z., Schütt O., Schiffmann F. (2020). CP2K: An Electronic
Structure and Molecular Dynamics Software Package - Quickstep: Efficient
and Accurate Electronic Structure Calculations. J. Chem. Phys.

[15] Perdew J. P., Burke K., Ernzerhof M. (1996). Generalized Gradient Approximation
Made Simple. Phys. Rev. Lett.

[16] Grimme S., Antony J., Ehrlich S., Krieg H. (2010). A Consistent and Accurate
Ab Initio Parametrization of Density Functional Dispersion Correction
(DFT-D) for the 94 Elements H-Pu. J. Chem. Phys.

[17] Dajnowicz S., Agarwal G., Stevenson J. M., Jacobson L. D., Ramezanghorbani F., Leswing K., Friesner R. A., Halls M. D., Abel R. (2022). High-Dimensional
Neural Network Potential for Liquid Electrolyte Simulations. J. Phys. Chem. B.

[18] Shayestehpour O., Zahn S. (2023). Efficient Molecular
Dynamics Simulations of Deep Eutectic Solvents
with First-Principles Accuracy Using Machine Learning Interatomic
Potentials. J. Chem. Theory Comput.

[19] Abraham M. J., Murtola T., Schulz R., Páll S., Smith J. C., Hess B., Lindahl E. (2015). GROMACS: High Performance
Molecular Simulations through Multi-Level Parallelism from Laptops
to Supercomputers. SoftwareX.

[20] Martínez L., Andrade R., Birgin E. G., Martínez J. M. (2009). PACKMOL:
A Package for Building Initial Configurations for Molecular Dynamics
Simulations. J. Comput. Chem.

[21] Anker S., McKechnie D., Mulheran P., Sefcik J., Johnston K. (2024). Assessment
of GAFF and OPLS Force Fields for Urea: Crystal and Aqueous Solution
Properties. Cryst. Growth Des.

[22] Berendsen, H. J. C. ; Postma, J. P. M. ; van Gunsteren, W. F. ; Hermans, J. Interaction Models for Water in Relation to Protein Hydration. In Intermolecular Forces; Springer, 1981; Vol. 14, pp. 331–342. DOI: 10.1007/978-94-015-7658-1_21

[23] Parrinello M., Rahman A. (1981). Polymorphic Transitions in Single
Crystals: A New Molecular
Dynamics Method. J. Appl. Phys.

[24] Martyna G.
J., Klein M. L., Tuckerman M. (1992). Nosé–Hoover Chains:
The Canonical Ensemble via Continuous Dynamics. J. Chem. Phys.

[25] Darden T., York D., Pedersen L. (1993). Particle Mesh
Ewald: An *N* ·log­(*N*) Method
for Ewald Sums in Large Systems. J. Chem. Phys.

[26] Kovács D. P., Moore J. H., Browning N. J., Batatia I., Horton J. T., Pu Y., Kapil V., Witt W. C., Magdău I.-B., Cole D. J., Csányi G. (2025). MACE-OFF: Short-Range Transferable
Machine Learning Force Fields for Organic Molecules. J. Am. Chem. Soc.

[27] Brehm M., Kirchner B. (2011). TRAVIS - A Free Analyzer
and Visualizer for Monte Carlo
and Molecular Dynamics Trajectories. J. Chem.
Inf. Model.

