# Supplementary material for: Zn2+ Coordination and Ligand-Exchange Dynamics in a ZnCl2‑Based Deep Eutectic Electrolyte from Machine-Learning Molecular Dynamics
Source: J Phys Chem B. 2026 Sep 26;130(40):10582–90. doi: 10.1021/acs.jpcb.6c04316 (PMC13656736; doi:10.1021/acs.jpcb.6c04316)
Supplement: Supplementary file 1 [file jp6c04316_si_001.pdf]

SUPPORTING MATERIAL

Zn^{2+} Coordination and Ligand-Exchange Dynamics in a $ZnCl_2$-Based Deep Eutectic Electrolyte from Machine-Learning Molecular Dynamics

Eudes Fileti,[1] Davi Fileti,[2] André P. L. de Carvalho,[2] Débora Ariana C. da Silva,[3] Shern Ren Tee,[4] Dinis O. Abranches,[5] João A. P. Coutinho[5]

[1] Instituto de Ciência e Tecnologia, Universidade Federal de São Paulo, 12247-014, São José dos Campos, SP, Brazil

[2] Institute of Mathematical Sciences and Computation, University of São Paulo, São Carlos, São Paulo, Brazil

[3] Institute of Science and Technology, Federal University of ABC, Santo Andre, São Paulo 09210-170, Brazil

[4] School of Environment and Science, Griffith University, Nathan, QLD, 4111, Australia

[5] CICECO − Aveiro Institute of Materials, Department of Chemistry, University of Aveiro, Aveiro 3810-193, Portugal

Sumário

S1. Computational Methods 3
S1.1 Generation of Reference Configurations by AIMD 3
S1.2 Dataset Construction 3
S1.3 Machine-Learning Force Field Training 4
S1.4 MLMD Simulation Protocol 5
S2. Model Validation 6
S2.1 DFT-MACE Energy and Force Agreement 6
S2.2 Effect of System Size on Structure 7
S3. Structural Analysis: Effect of Temperature 9
S4. Diffusion Coefficients and Activation Energies 11
S5. Residence-Time Analysis: Methodology 12
S6. Structural Convergence of the Production Trajectories 13
References 15

S1. Computational Methods

S1.1 Generation of Reference Configurations by AIMD

Ab initio molecular dynamics (AIMD) simulations were performed using the CP2K software package.[1] Electronic structure calculations were carried out within density functional theory (DFT) using the PBE functional augmented with Grimme's D3 dispersion correction (PBE-D3),[2] chosen for its proven reliability in describing non-covalent interactions, ion-dipole coordination, and hydrogen bonding in ionic liquids and eutectic systems. Core electrons were treated using Goedecker-Teter-Hutter (GTH) pseudopotentials,[3] and the valence electronic structure was expanded in a double-ζ MOLOPT-DZVP-SR-GTH basis set.[4] Plane-wave and relative cutoffs were set to 350 Ry and 40 Ry, respectively, and the SCF convergence criterion was fixed at 10^{-6}. All simulations were performed in the NVT ensemble using the QUICKSTEP module of CP2K.[1]

To maximize conformational diversity while minimizing temporal correlations inherent to short AIMD trajectories, a multi-seed sampling strategy was adopted. Ten independent simulations of 6 ps each (1 ps equilibration followed by 5 ps production) were initiated from structurally distinct starting configurations generated by classical molecular dynamics using GROMACS.[5] Five of these trajectories were run at 300 K, three at 400 K, and two at 500 K. The elevated temperatures were employed not to represent physically stable states of the electrolyte, which lies above its estimated thermal stability range, but rather to enhance conformational sampling of the potential energy surface. Approximately 1,000 frames were extracted from each trajectory via the CP2K REFTRAJ module, yielding a total raw dataset of ~10,000 configurations (~5,000 at 300 K, ~3,000 at 400 K, and ~2,000 at 500 K).

S1.2 Dataset Construction

Single-point energy and force recalculations were performed on the sampled configurations at an elevated level of theory (PBE-D3, with a plane-wave cutoff of 650 Ry) to obtain the reference data used for model training. This two-level protocol provides structural diversity and accurate energetics with a practical balance between sampling efficiency and reference quality.

To construct a balanced training set, configurations were subsampled from each temperature subset using uniform strides, yielding approximately 2,800 training frames (1,300 at 300 K; 1,000 at 400 K; and 500 at 500 K). An independent test set of 600 configurations (200 per temperature) was reserved from held-out trajectories (one trajectory per temperature group), ensuring complete independence between training and evaluation data at the level of initial configurations rather than individual frames. A temperature-dependent weighting scheme was applied during training, with weights of 1.0, 0.8, and 0.3 assigned to the 300, 400, and 500 K subsets, respectively, reflecting the physical relevance of near-ambient conditions for the target applications. The training

set was further partitioned into training (90%, ~2,500 configurations) and validation (10%, ~270 configurations) subsets, with the split performed randomly after shuffling to avoid temperature-based clustering.

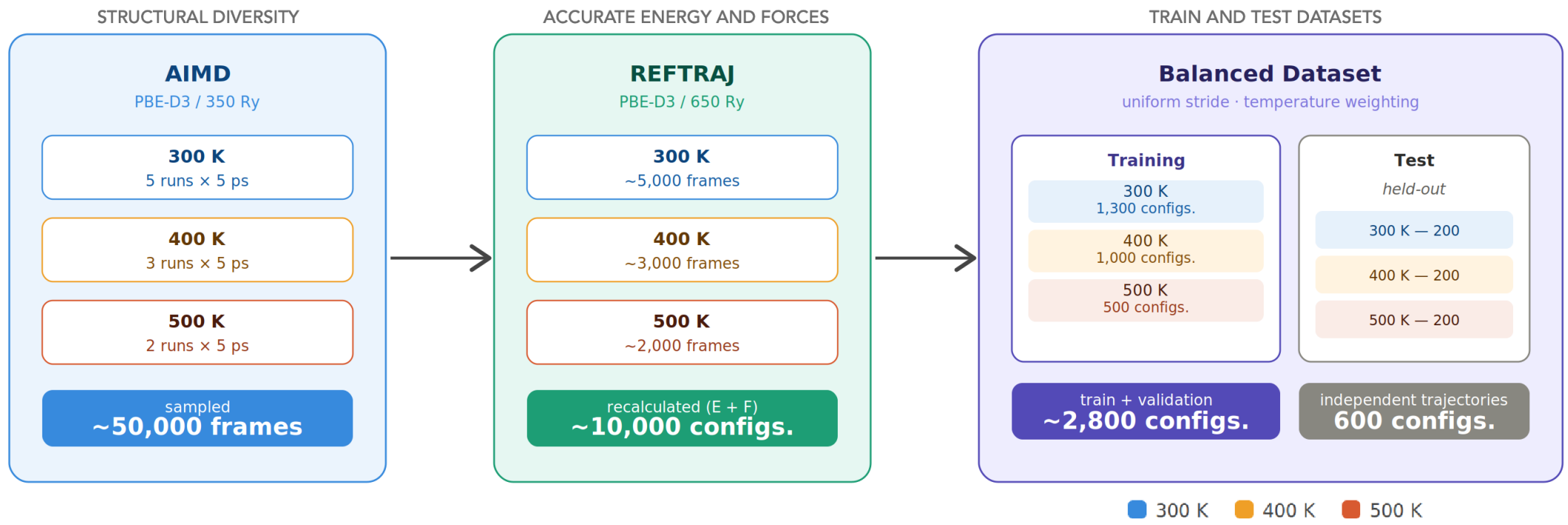

Figure S1: Dataset construction pipeline. AIMD simulations at three temperatures (300, 400, and 500 K) generated ~50,000 structural configurations, from which ~10,000 were selected for high-level single-point energy and force calculations (REFTRAJ). The final dataset comprises 2,800 training and 600 test configurations, balanced across temperatures.

S1.3 Machine-Learning Force Field Training

The machine-learning force field (MLFF) was developed by fine-tuning the MACE-MP-0 small foundation model[6] on the DFT reference dataset described above. MACE (Message-passing Atomic Cluster Expansion) is an equivariant graph neural network that combines the Atomic Cluster Expansion framework[7] with higher-order message-passing operations, enforcing E(3) symmetry by construction.[6] Fine-tuning from a foundation model pre-trained on ~150,000 Materials Project structures[8] was preferred over training from scratch, as it provides well-initialized representations for all six elements in the system (C, H, N, O, Cl, Zn) and requires significantly less reference data to achieve convergence, a strategy increasingly adopted for liquid-phase MLFFs.[9]

Fine-tuning was performed using the aMACEing toolkit.[9] The model architecture (2 message-passing layers, correlation order 3, spherical harmonics up to $l = 3$, 522,394 parameters) was inherited from the foundation model, with a radial cutoff of 6.0 Å. Training employed the Adam optimizer (learning rate 0.01, gradient clipping 10.0) with a weighted loss function combining energy (weight 1.0) and force (weight 10.0) terms. Temperature-dependent configuration weights of 1.0, 0.8, and 0.3 were assigned to the 300, 400, and 500 K subsets, respectively. Training ran for 200 epochs on an NVIDIA Tesla V100 GPU using float64 precision.

The final model achieved force RMSEs of 24.9 and 25.3 meV/Å on the training and validation sets, respectively, with energy RMSEs of 1.8 meV/atom in both cases. These values are consistent with MACE-based MLFFs reported for related electrolyte systems[10] and compare favorably with DeePMD models for ionic liquids, where force

errors of 30-80 meV/Å are typical.[11] The negligible train/validation discrepancy confirms adequate dataset diversity and the absence of overfitting.

S1.4 MLMD Simulation Protocol

Machine-learning molecular dynamics (MLMD) simulations were carried out using LAMMPS[12] interfaced with the trained MACE potential via the ACEsuit fork of LAMMPS,[13] which provides native support for the pair_style mace interaction. All simulations employed a timestep of 1 fs and three-dimensional periodic boundary conditions. Three system sizes were constructed from the original AIMD cell to assess finite-size effects on structural and dynamical properties (Section S2.2): small (491 atoms, 17.9 Å), medium (982 atoms, 22.6 Å), and large (1964 atoms, 28.5 Å). The medium box was adopted for all production simulations as the best compromise between structural accuracy and computational cost; the large box, while useful for size-convergence testing, proved too computationally demanding for the 1 ns, five-temperature production protocol used for the dynamical analyses presented in the main text.

Each simulation followed a three-stage protocol. First, a conjugate-gradient energy minimization was performed (force and energy convergence thresholds of 10^{-4} eV/Å and 10^{-4} eV, respectively; maximum 10,000 force evaluations) to remove any residual strain in the initial configuration. Second, an equilibration run of 50 ps was performed in the NVT ensemble (Section S2.2) using a Nosé-Hoover thermostat with a damping time of 0.1 ps, with atomic velocities initialized from a Maxwell-Boltzmann distribution at the target temperature. Third, production runs of 1 ns were carried out under the same NVT conditions, with atomic positions, velocities, and element identities written every 50 fs for subsequent analysis. A linear momentum-zeroing correction was applied every 1,000 steps to prevent center-of-mass drift.

Production simulations were performed independently at five temperatures (300, 325, 350, 375, and 400 K), spanning the physically accessible liquid range of the $ZnCl_2$:urea:H_2O electrolyte at the 1:3:1 molar composition. Structural properties, including radial distribution functions (RDFs) and Zn^{2+} coordination numbers, were computed using the TRAVIS program.[14] Translational diffusion coefficients were extracted from the mean-square displacement (MSD) of each species in the diffusive regime, and the activation energy for diffusion was obtained from Arrhenius fits to the temperature-dependent data. All analyses were performed on the full production trajectories after discarding the equilibration period.

S2. Model Validation

S2.1 DFT-MACE Energy and Force Agreement

The MACE model demonstrates excellent agreement with the reference DFT calculations across the full test set (Figure S2). For the potential energies, the model accurately reproduces the DFT reference values over the entire energy range (±25 meV/atom), yielding an energy RMSE of 1.9 meV/atom, well below the sub-5 meV/atom threshold commonly considered sufficient for production-quality machine-learning force fields. The absence of systematic deviations or curvature indicates that the model correctly captures the underlying energy landscape across all sampled configurations. For the atomic forces, the MACE model achieves a force RMSE of 31 meV/Å, also within the accuracy range typically regarded as suitable for MLMD simulations of molecular systems. The force components span approximately ±8 eV/Å, reflecting the broad variety of local atomic environments sampled at different temperatures; most force components are concentrated within the ±2 eV/Å region, with a smooth distribution extending toward higher-force configurations and no systematic deviation from the diagonal, confirming the robustness and consistency of the model predictions.

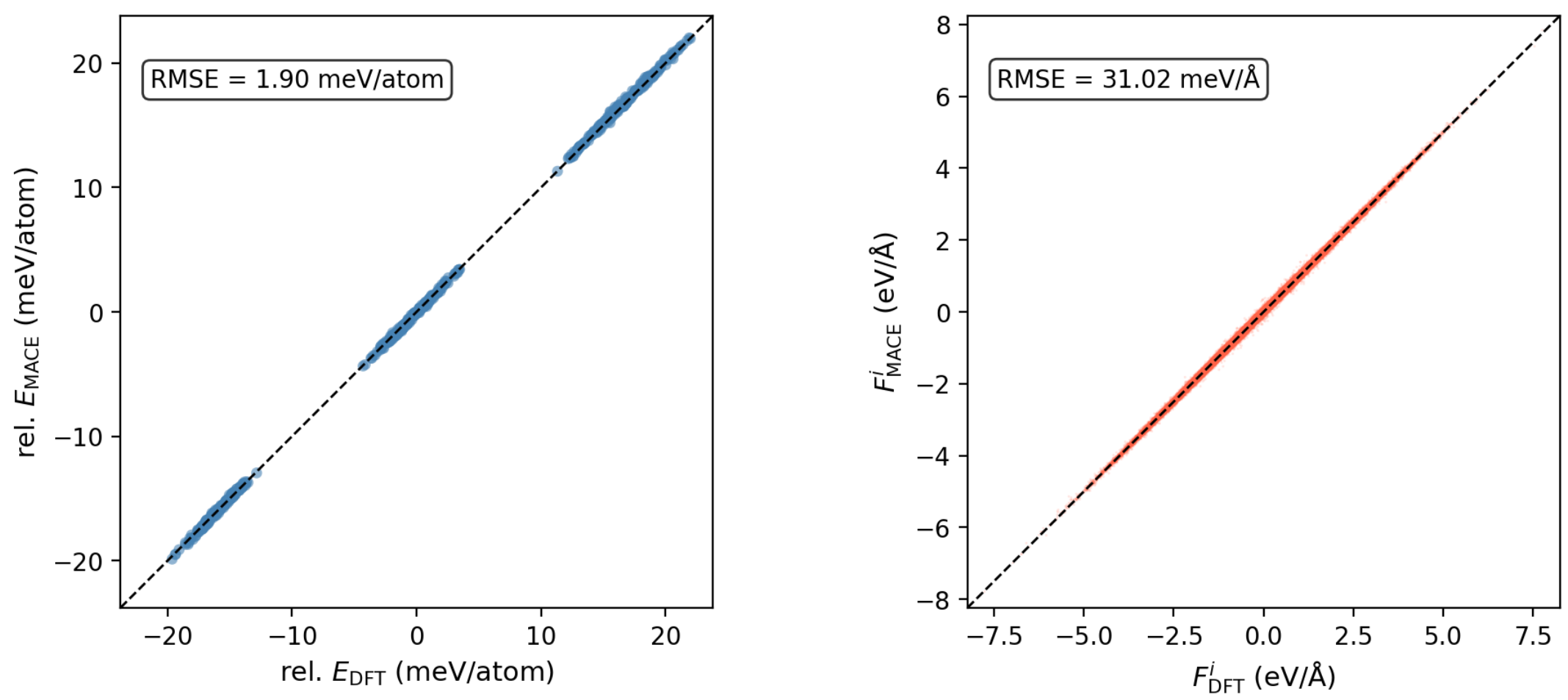

Figure S2: Parity plots of potential energy per atom and atomic force components predicted by the MACE model with PBE-D3 reference values for the independent test set of 600 configurations. Dashed lines indicate perfect agreement (1:1 diagonal).

S2.2 Effect of System Size on Structure

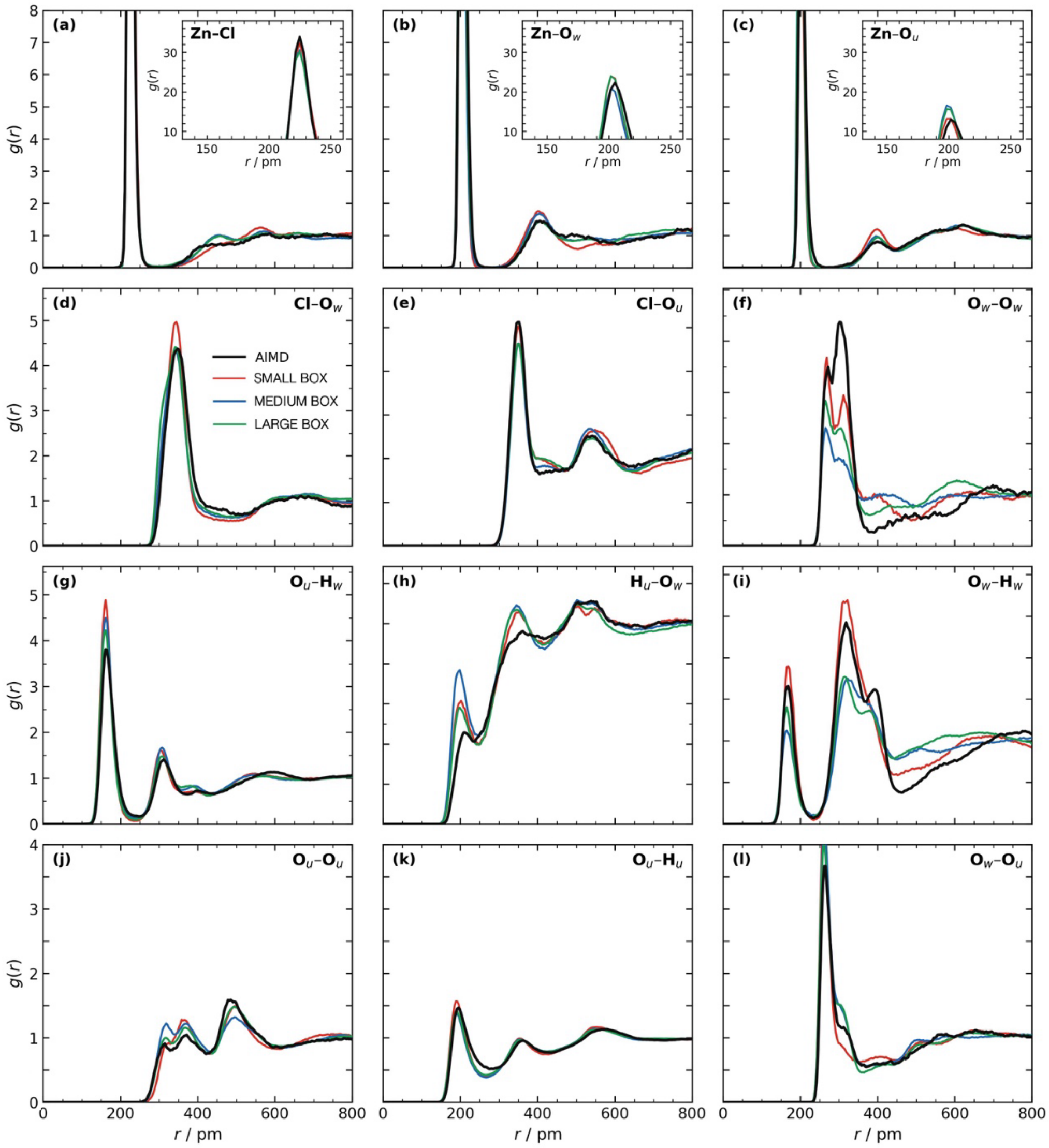

Figure S3A: Radial distribution functions for the $ZnCl_2$:urea:H_2O (1:3:1) deep eutectic electrolyte at 300 K obtained from ab initio molecular dynamics (AIMD, black, 491 atoms) and machine-learning molecular dynamics using the MACE force field (MLMD) with different simulation box sizes: small (red, 491 atoms), medium (blue, 982 atoms), and large (green, 1964 atoms). Panels (a)-(c) show Zn^{2+}-centered pairs, with insets highlighting the first coordination-shell peak. Panels (d)-(f) show Cl^--centered and water oxygen-oxygen correlations; panels (g)-(l) present correlations involving urea and water species.

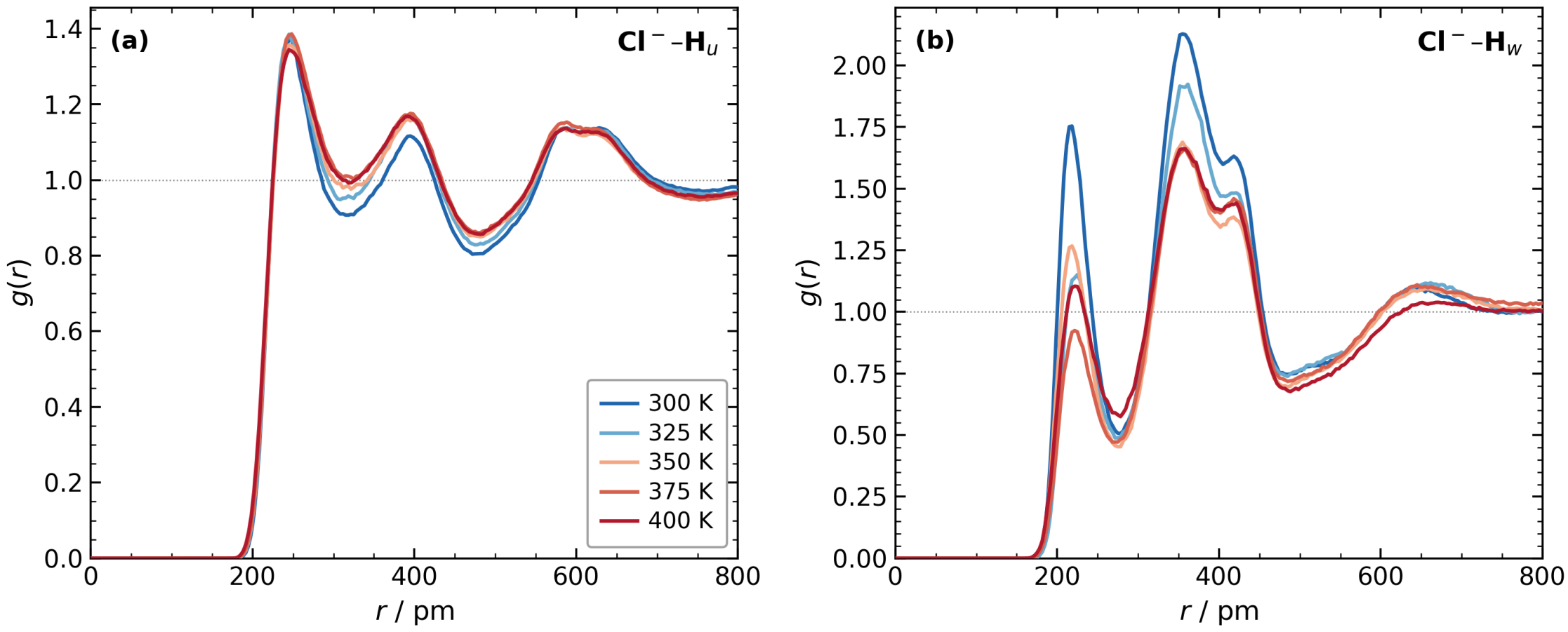

Figure S3B: Chloride-hydrogen RDFs for the $ZnCl_2$:urea:H_2O (1:3:1) electrolyte from 1 ns MLMD trajectories (medium box) at 300-400 K. (a) Cl^--H(urea) and (b) Cl^--H(water). The first peaks, near 245 and 220 pm respectively, correspond to N-H···Cl^- and O-H···Cl^- hydrogen bonds; the outer features arise from the non-bonded hydrogens of the same donor molecules. The Cl^--H(urea) first shell is essentially temperature-invariant, whereas the Cl^--H(water) correlation weakens with increasing temperature.

The radial distribution functions obtained from AIMD and MLMD simulations show overall excellent agreement, demonstrating that the MACE potential accurately captures the local and intermediate-range structural organization of this complex hydrogen-bonded ionic system. The Zn-centered correlations (Zn-Cl, Zn-Ow, Zn-Ou) exhibit nearly identical first-shell peak positions and very similar peak intensities across all box sizes, confirming that the MACE model reproduces the Zn^{2+} coordination environment and the balance between chloride, water, and urea interactions around the cation regardless of system size. The Cl-centered RDFs and the correlations involving urea and water display similarly consistent structural features, including the position and general shape of second-shell organization; the hydrogen-bond-related RDFs (Ou-Hw, Ow-Hw) likewise confirm that the MACE force field captures the hydrogen-bond network and solvent structuring with good accuracy.

Some differences are observed in the relative intensities and widths of specific peaks, especially for broader second-shell correlations and for RDFs involving water molecules (Ow-Ow, Ow-Hw), where MLMD predicts a slightly less structured liquid than AIMD. Variations among the small, medium, and large box simulations do not follow a clear monotonic trend with system size: for several RDFs, peak heights fluctuate non-systematically as the box size increases, indicating that the remaining discrepancies reflect intrinsic statistical variability rather than a genuine finite-size effect. These differences remain modest and do not alter the overall structural interpretation.

Among the MLMD systems, the medium box provides an excellent compromise between structural accuracy and computational efficiency, closely reproducing the AIMD reference across essentially all pair correlations. This validates the use of the medium box for the production simulations at elevated temperatures discussed in the main text.

S3. Structural Analysis: Effect of Temperature

The radial distribution functions computed from MLMD at five temperatures spanning 300-400 K reveal a structurally robust electrolyte in which the first coordination shell of Zn^{2+} is remarkably insensitive to thermal perturbation. The Zn-Cl RDF exhibits a sharp, intense first peak at approximately 225 pm that remains essentially invariant in position across the entire temperature range, with only a modest, monotonic reduction in peak height from ~31 at 300 K to ~28 at 400 K; the corresponding coordination number decreases very slightly from 2.04 to 2.00. This near-invariance indicates that Zn-Cl coordination is energetically robust and not disrupted by the thermal energy available in this range. The Zn-Ow and Zn-Ou first-shell peaks (~203 and ~201 pm, respectively) similarly show negligible positional shift with temperature, though their heights decrease modestly. The Zn-Ow coordination number fluctuates non-monotonically between 0.57 and 0.66, while Zn-Ou increases from 1.35 at 300 K to 1.48 at 400 K, suggesting a subtle but systematic enhancement of urea coordination around Zn^{2+} at elevated temperatures.

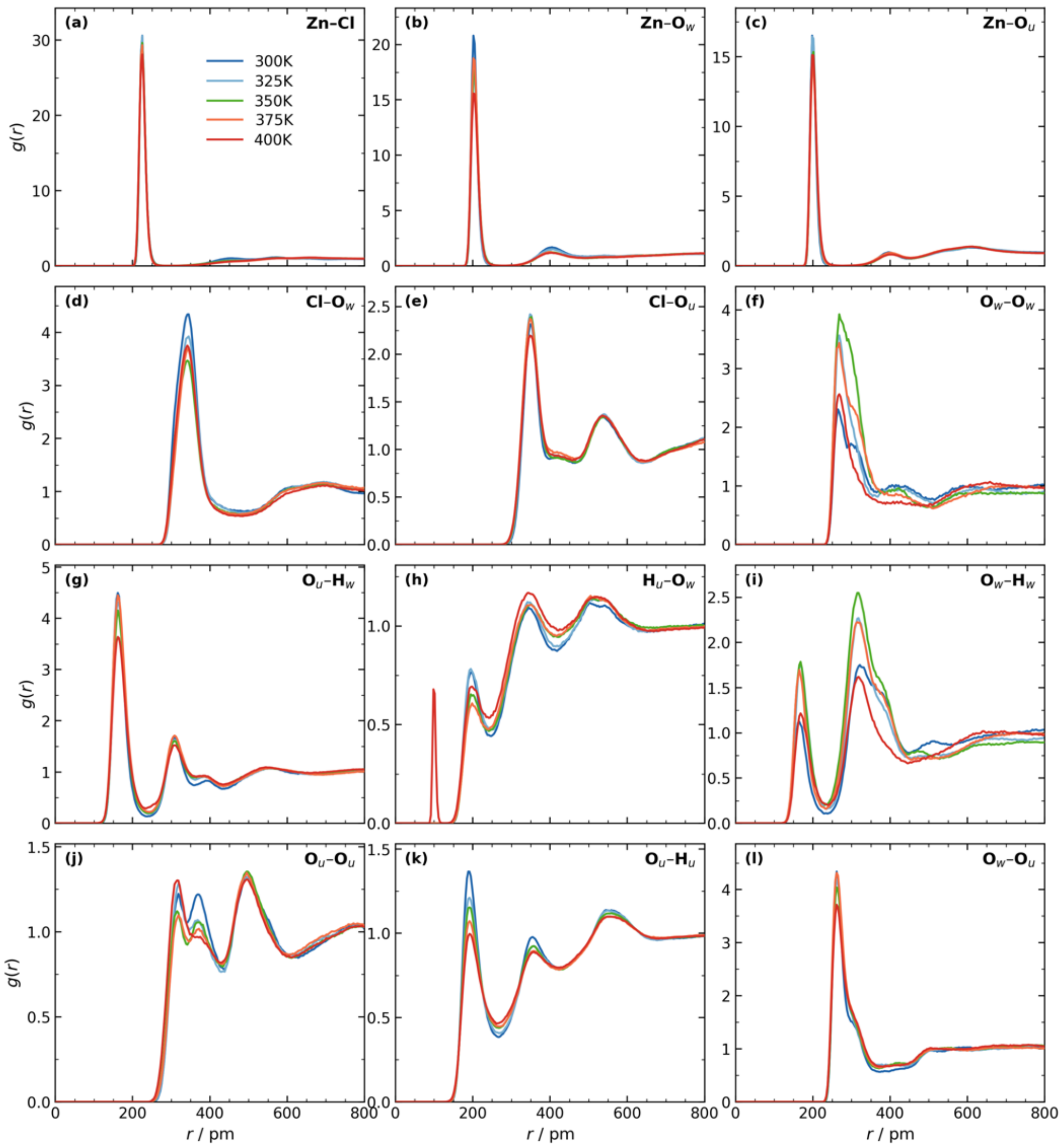

Figure S4: Radial distribution functions for all relevant pair correlations in the $ZnCl_2$:urea:H_2O deep eutectic electrolyte computed from MLMD at five temperatures (300-400 K, medium box, 982 atoms). Panels (a)-(c) show Zn^{2+}-centered pairs; panels (d)-(f) show Cl^--centered and water oxygen-oxygen correlations; panels (g)-(i) show hydrogen-bond-related correlations; panels (j)-(l) show urea-urea and water-urea correlations. Colors indicate temperature from cold (blue, 300 K) to warm (red, 400 K).

The Cl^--centered correlations show more pronounced thermal sensitivity. The Cl-Ow first peak near 344 pm decreases systematically in height from ~4.4 to ~3.7 with increasing temperature, accompanied by broadening and flattening of the second shell, indicative of progressive disruption of the hydrogen-bond-mediated hydration layer around the chloride anion. The Cl-Ou peak near 351 pm follows a similar but weaker trend. CN(Cl-Ow) and CN(Cl-Ou) both increase moderately with temperature, from 1.53 to 1.27 and from 1.70 to 2.01, respectively, though the latter is non-monotonic at intermediate temperatures, reflecting the competitive and dynamic nature of urea and water coordination around Cl^-.

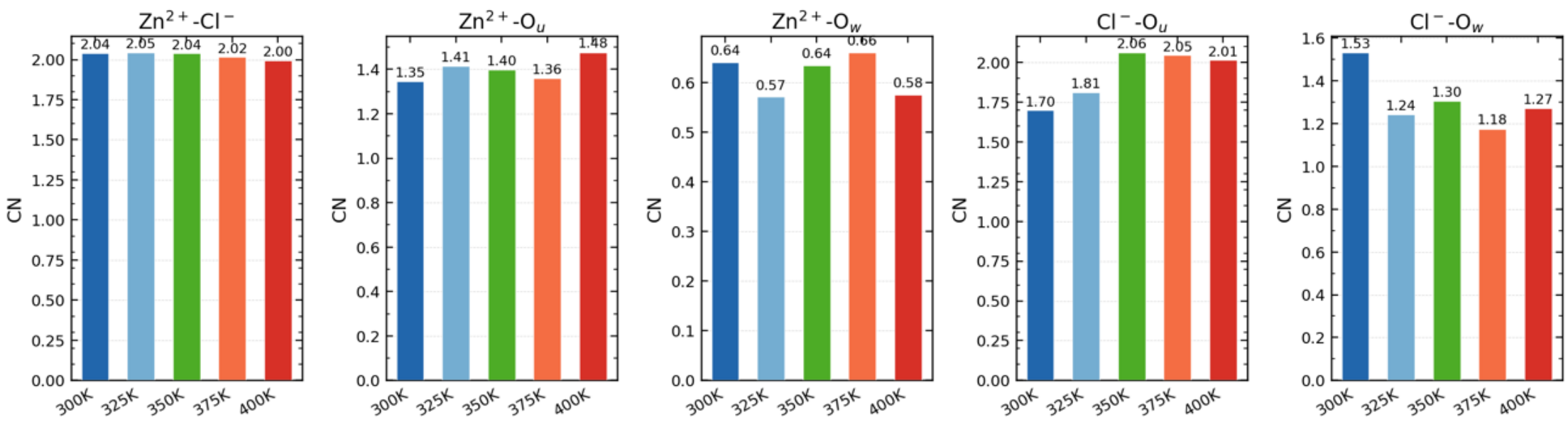

Figure S5: Coordination numbers for the five most relevant ion-solvent pairs as a function of temperature. Values extracted from MLMD RDF integrals (medium box, 982 atoms) up to the first-shell minimum. The near-constancy of Zn^{2+}-Cl^- coordination contrasts with the greater variability observed for Cl^--solvent interactions, highlighting the differential thermal stability of cation and anion solvation shells.

S4. Diffusion Coefficients and Activation Energies

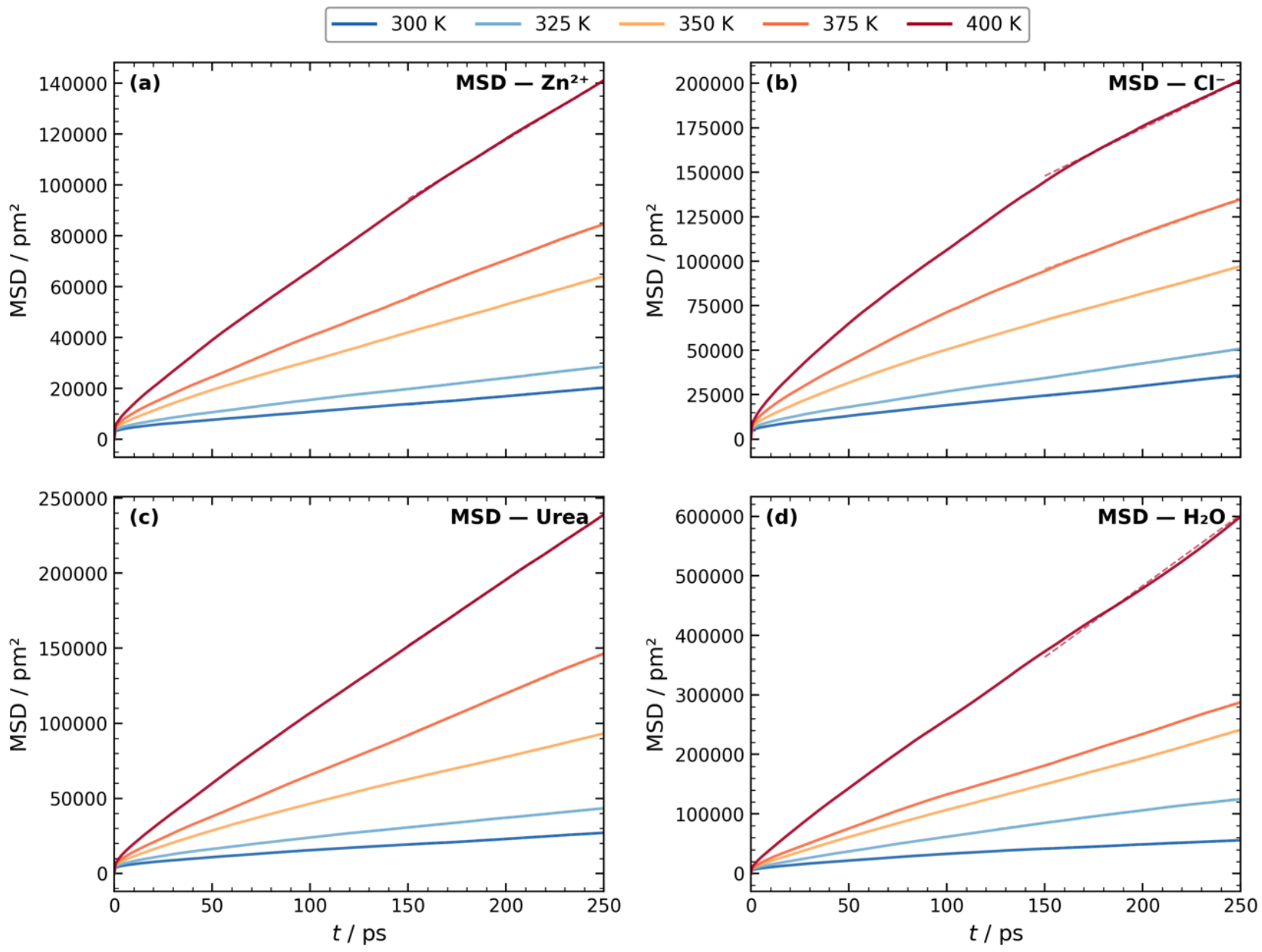

Figure S6: Mean-square displacement (MSD) curves for all four constituent species of the $ZnCl_2$:urea:H_2O deep eutectic electrolyte: (a) Zn^{2+}, (b) Cl^-, (c) urea, and (d) H_2O, at five temperatures (300-400 K). Solid lines: MSD; dashed lines: linear fits in the diffusive regime. All curves reach a well-defined linear regime within the 1 ns simulation window, validating the extracted diffusion coefficients.

Table S1: Self-diffusion coefficients D (in 10^{-12} m2 s-1) and Arrhenius activation energies Ea (kJ mol-1) for all four constituent species of the $ZnCl_2$:urea:H_2O deep eutectic electrolyte, computed from 1 ns MLMD trajectories (medium box, 982 atoms).

Species	D / 10^{-12} m^2 s^{-1}					E_a / kJ mol^{-1}
	300 K	325 K	350 K	375 K	400 K	
Zn^{2+}	11.5	14.6	36.8	47.9	77.6	19.9
Cl^-	19.8	27.2	51.4	65.4	89.3	15.6
Urea	13.7	21.4	51.5	90.4	145.3	24.6
H_2O	23.0	68.7	152.4	180.3	399.3	26.9

The temperature dependence of D was analyzed within the Arrhenius framework:

$$D(T) = D_0 \exp\left(-\frac{E_a}{RT}\right)$$

Linearizing this expression yields:

$$\ln D = -\frac{E_a}{R}\cdot\frac{1}{T} + \ln D_0 = -\frac{E_a}{R \times 1000}\cdot\frac{1000}{T} + \ln D_0$$

so that a plot of lnD versus 1000/T has slope $-E_a/(R \times 1000)$, from which the activation energy is recovered as:

$$E_a\ [\mathrm{kJ\ mol^{-1}}] = -\mathrm{slope} \times R \times 1000 = -\mathrm{slope} \times 8.314$$

where R = 8.314 × 10^{-3} kJ mol^{-1} K^{-1}. The Arrhenius plot is shown in Figure 4c of the main text.

S5. Residence-Time Analysis: Methodology

The diffusion coefficients and activation energies discussed in Section S4 characterize ion transport at the macroscopic level but do not reveal the microscopic exchange dynamics governing how long individual ligands remain bound within the Zn^{2+} first coordination shell. To address this, the residence time τ of each coordinating species (Cl^-, O(urea), O(water)) was computed via the dimer existence autocorrelation function (DACF), as implemented in TRAVIS. The DACF C(t) is defined as the normalized probability that a given pair, identified as associated at time t = 0 based on a distance criterion $r < r_{cut}$, remains continuously associated at time t. The residence time τ is then extracted as the integral of the fitted poly-exponential decay:

$$C(t) = \sum_i c_i \exp\left(-\frac{t}{\tau_i}\right) \quad ; \quad \tau_{\mathrm{total}} = \sum_i c_i\ \tau_i$$

with up to three exponential components. The cutoff distances were determined from the first minima of the respective radial distribution functions: r_{cut}(Zn-Cl) = 295 pm, r_{cut}(Zn-Ou) = 250 pm, and r_{cut}(Zn-Ow) = 260 pm. A correlation depth of 5000 frames (500 ps) was used at all temperatures.

Long residence times imply strongly bound, slowly exchanging ligands, which suppress ionic mobility and contribute to the high viscosity characteristic of deep eutectic electrolytes; conversely, the activation energy Ea for ligand exchange provides a thermodynamic measure of the kinetic barrier to desolvation, with direct implications for ion transport mechanisms and electrolyte performance. The temperature dependence of τ was analyzed within the same Arrhenius framework used for diffusion:

$$\tau(T) \ = \ \tau_0 \ \exp\left(\frac{E_a}{RT}\right) \quad ; \quad \ln \tau = \frac{E_a}{R} \cdot \frac{1}{T} + \mathrm{const}$$

where E_a is the activation energy for ligand exchange, obtained from the slope of ln τ versus 1000/T by linear regression. Results of this analysis are presented and discussed in the main text (Figure 5).

S6. Structural Convergence of the Production Trajectories

Because the production simulations were carried out in the canonical ensemble over 1 ns, we verified that the local structure around Zn^{2+} is stationary over the course of each trajectory rather than drifting systematically. For every temperature, two structural descriptors were monitored as a function of simulation time: the first-shell coordination numbers CN(Zn-Cl) and CN(Zn-O) (O = urea + water), and the number of independent ionic aggregates N_{agg}, defined as the connected components of the Zn-Cl contact network (two Zn^{2+} centers assigned to the same aggregate when they share a bridging chloride within the first-shell cutoff). Figure S7a shows the time evolution of these quantities at all five temperatures.

To quantify residual drift, each trajectory was divided into two halves and the relative change in the block-averaged descriptors between the first and second halves was computed (Table S7b). At 375 and 400 K the drift in all three descriptors is below 2%, indicating a fully equilibrated, stationary local structure; at 325 K the drift is likewise small (<2%). At 300 and 350 K a slow, monotonic evolution is observed, with residual drift of 3-6%, corresponding to the gradual replacement of first-shell chloride by urea/water oxygen. This residual drift is consistent with the long ligand residence times reported in the main text; τ(Zn-Cl) exceeds the 1 ns window at these temperatures; and with the high viscosity expected for the DES at lower temperature; it therefore reflects a genuine, slow physical relaxation of the coordination shell rather than incomplete numerical equilibration. Because chloride rearrangement is the slowest process in the system, descriptors that do not depend on it converge substantially faster, and the dynamical properties reported at 375-400 K, where all descriptors are converged to within 2%, are unaffected by this low-temperature relaxation.

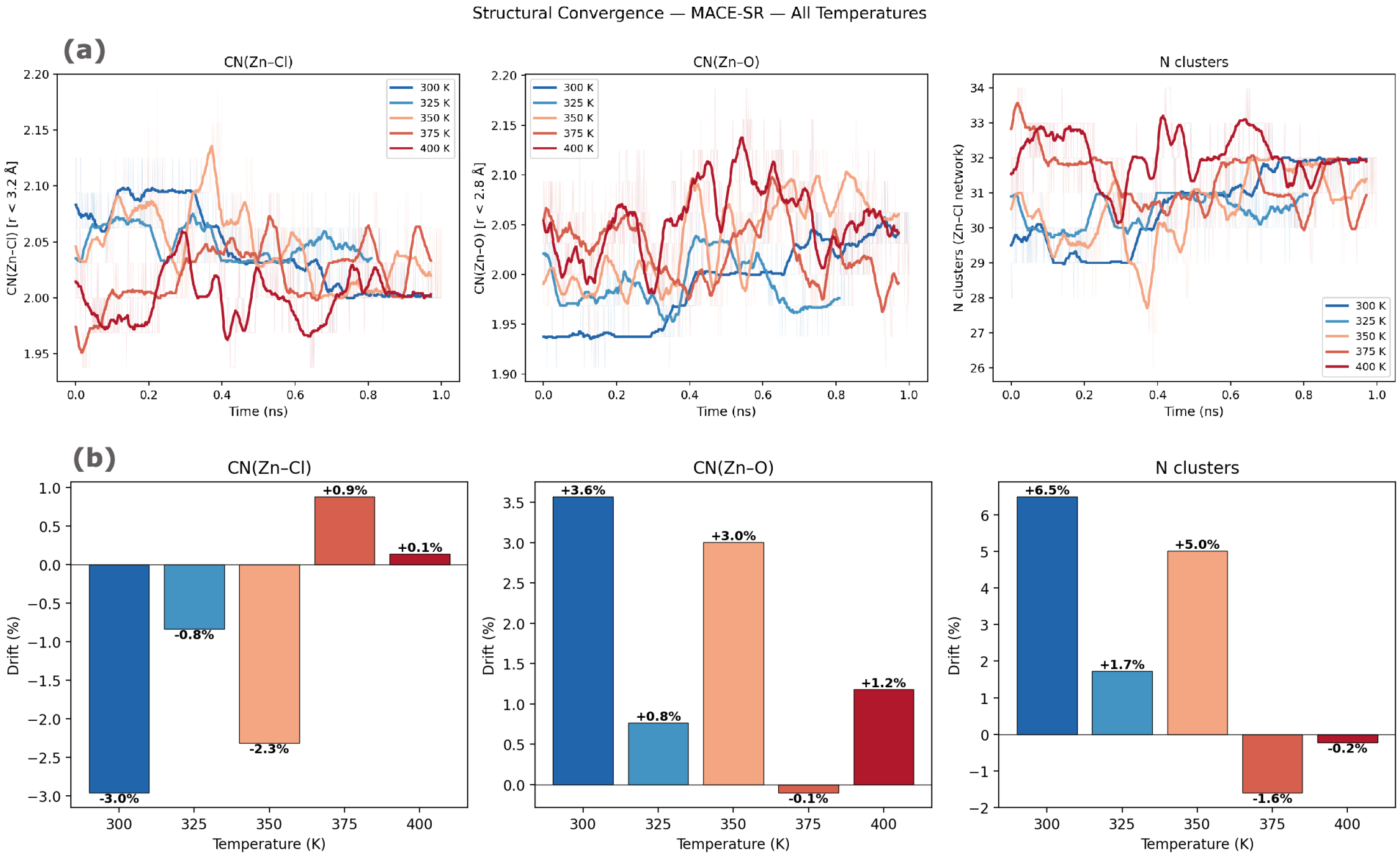

Figure S7: Structural convergence of the 1 ns MLMD production trajectories for the $ZnCl_2$:urea:H_2O (1:3:1) deep eutectic electrolyte at the five temperatures studied (300-400 K). (a) Time evolution of the first-shell coordination numbers CN(Zn-Cl) (r < 3.2 Å) and CN(Zn-O) (urea + water, r < 2.8 Å) and of the number of independent ionic aggregates N_{agg} (connected components of the Zn-Cl contact network), shown as running block averages (bold lines) over the instantaneous values (light traces). The descriptors are stationary at 375 and 400 K; at 300 K, CN(Zn-O) exhibits a step-like increase near 0.3 ns that marks a discrete ligand-exchange event, illustrating that the residual evolution at the lowest temperatures reflects slow physical chloride/oxygen exchange in the Zn^{2+} first shell rather than numerical drift. (b) Relative drift of the three descriptors between the first and second halves of each trajectory. The opposite signs of the CN(Zn-Cl) and CN(Zn-O) drift at 300, 325 and 350 K (chloride decreasing, oxygen increasing) reflect this same coordinated exchange; all descriptors are converged to within ~2% at 375 and 400 K, so that the dynamical properties reported in the main text are unaffected by the low-temperature relaxation.

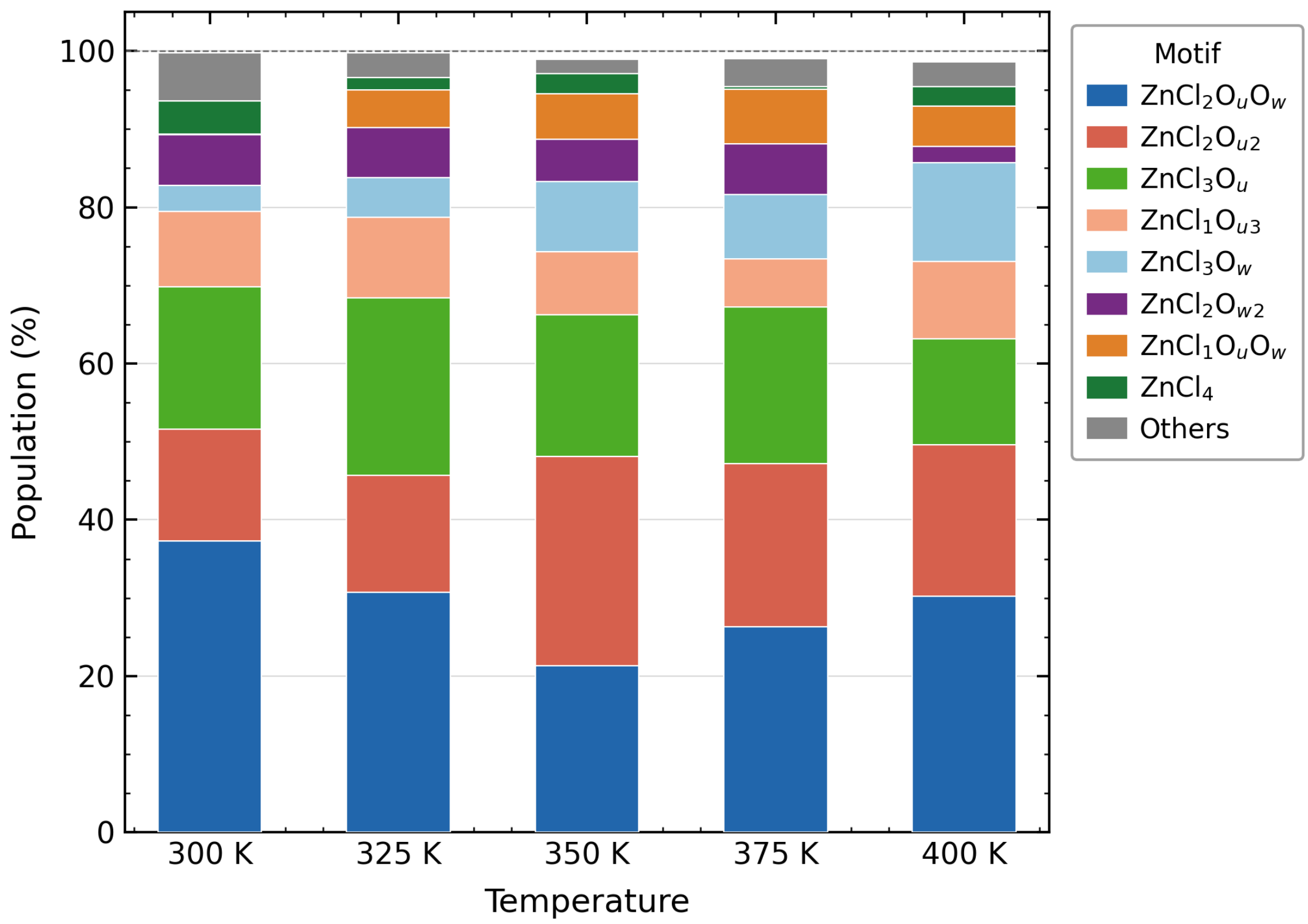

Figure S8: Temperature dependence of the Zn^{2+} first-shell coordination motif populations in the $ZnCl_2$:urea:H_2O (1:3:1) deep eutectic electrolyte, computed from 1 ns MLMD trajectories (medium box, 982 atoms) at five temperatures (300-400 K). Each Zn^{2+} center was assigned at every trajectory frame to a motif ZnCl_x O(U)_y O(W)_z defined by the number of Cl^- (cutoff 2.95 Å), O(urea) (2.50 Å), and O(water) (2.60 Å) donors in its first shell; only motifs with total coordination number CN = 4 are shown (93-97% of all frames). Motifs with population < 4% at all temperatures are grouped as "Others".

References

(1) Kühne, T. D.; Iannuzzi, M.; Del Ben, M.; Rybkin, V. V.; Seewald, P.; Stein, F.; Laino, T.; Khaliullin, R. Z.; Schütt, O.; Schiffmann, F.; Golze, D.; Wilhelm, J.; Chulkov, S.; Bani-Hashemian, M. H.; Weber, V.; Borštnik, U.; Taillefumier, M.; Jakobovits, A. S.; Lazzaro, A.; Pabst, H.; Müller, T.; Schade, R.; Guidon, M.; Andermatt, S.; Holmberg, N.; Schenter, G. K.; Hehn, A.; Bussy, A.; Belleflamme, F.; Tabacchi, G.; Glöß, A.; Lass, M.; Bethune, I.; Mundy, C. J.; Plessl, C.; Watkins, M.; VandeVondele, J.; Krack, M.; Hutter, J. CP2K: An Electronic Structure and Molecular Dynamics Software Package - Quickstep: Efficient and Accurate Electronic Structure Calculations. *J. Chem. Phys.* **2020**, *152* (19). https://doi.org/10.1063/5.0007045.

(2) Grimme, S.; Antony, J.; Ehrlich, S.; Krieg, H. A Consistent and Accurate *Ab Initio* Parametrization of Density Functional Dispersion Correction (DFT-D) for the 94

Elements H-Pu. *J. Chem. Phys.* **2010**, *132* (15). https://doi.org/10.1063/1.3382344.
(3) Goedecker, S.; Teter, M.; Hutter, J. Separable Dual-Space Gaussian Pseudopotentials. *Phys. Rev. B* **1996**, *54* (3), 1703–1710. https://doi.org/10.1103/PhysRevB.54.1703.
(4) VandeVondele, J.; Hutter, J. Gaussian Basis Sets for Accurate Calculations on Molecular Systems in Gas and Condensed Phases. *J. Chem. Phys.* **2007**, *127* (11). https://doi.org/10.1063/1.2770708.
(5) Abraham, M. J.; Murtola, T.; Schulz, R.; Páll, S.; Smith, J. C.; Hess, B.; Lindahl, E. GROMACS: High Performance Molecular Simulations through Multi-Level Parallelism from Laptops to Supercomputers. *SoftwareX* **2015**, *1–2*, 19–25. https://doi.org/10.1016/j.softx.2015.06.001.
(6) Batatia, I. ; P. K. D. ; S. G. N. C. ; O. C. ; C. G. MACE: Higher Order Equivariant Message Passing Neural Networks for Fast and Accurate Force Fields. . *Adv. Neural Inf. Process. Syst.* **2022**, *35*, 11423–11436.
(7) Drautz, R. Atomic Cluster Expansion for Accurate and Transferable Interatomic Potentials. *Phys. Rev. B* **2019**, *99* (1), 014104. https://doi.org/10.1103/PhysRevB.99.014104.
(8) Jain, A.; Montoya, J.; Dwaraknath, S.; Zimmermann, N. E. R.; Dagdelen, J.; Horton, M.; Huck, P.; Winston, D.; Cholia, S.; Ong, S. P.; Persson, K. The Materials Project: Accelerating Materials Design Through Theory-Driven Data and Tools. In *Handbook of Materials Modeling*; Springer International Publishing: Cham, 2020; pp 1751–1784. https://doi.org/10.1007/978-3-319-44677-6_60.
(9) Xiao, Z.; Dai, X.; Zhu, J.; Liu, D.; Liu, L.; Liu, X.; Li, Y.; Qian, Z.; Wang, R. Hydrogen Bond Competition Optimizing Aqueous Zn Ion Solvation and (002) Interfacial Deposition with Ultralong Stability. *Adv. Funct. Mater.* **2025**, *35* (23). https://doi.org/10.1002/adfm.202424860.
(10) Brahma, D.; Avula, N. V. S.; Balasubramanian, S. Hydration Environment of Complexes and Their Dynamics in Aqueous Zinc Chloride Solutions. *J. Phys. Chem. B* **2026**. https://doi.org/10.1021/acs.jpcb.6c01759.
(11) Shayestehpour, O.; Zahn, S. Efficient Molecular Dynamics Simulations of Deep Eutectic Solvents with First-Principles Accuracy Using Machine Learning Interatomic Potentials. *J. Chem. Theory Comput.* **2023**, *19* (23), 8732–8742. https://doi.org/10.1021/acs.jctc.3c00944.
(12) Thompson, A. P.; Aktulga, H. M.; Berger, R.; Bolintineanu, D. S.; Brown, W. M.; Crozier, P. S.; in 't Veld, P. J.; Kohlmeyer, A.; Moore, S. G.; Nguyen, T. D.; Shan, R.; Stevens, M. J.; Tranchida, J.; Trott, C.; Plimpton, S. J. LAMMPS - a Flexible Simulation Tool for Particle-Based Materials Modeling at the Atomic, Meso, and Continuum Scales. *Comput. Phys. Commun.* **2022**, *271*, 108171. https://doi.org/10.1016/j.cpc.2021.108171.
(13) Kovács, D. P.; Moore, J. H.; Browning, N. J.; Batatia, I.; Horton, J. T.; Pu, Y.; Kapil, V.; Witt, W. C.; Magdău, I.-B.; Cole, D. J.; Csányi, G. MACE-OFF: Short-Range Transferable Machine Learning Force Fields for Organic Molecules. *J. Am. Chem. Soc.* **2025**, *147* (21), 17598–17611. https://doi.org/10.1021/jacs.4c07099.
(14) Brehm, M.; Kirchner, B. TRAVIS - A Free Analyzer and Visualizer for Monte Carlo and Molecular Dynamics Trajectories. *J. Chem. Inf. Model.* **2011**, *51* (8), 2007–2023. https://doi.org/10.1021/ci200217w.
